# Recent Advances in Bimetallic Nanoporous Gold Electrodes for Electrochemical Sensing

**DOI:** 10.3390/nano13182515

**Published:** 2023-09-08

**Authors:** Md. Shafiul Islam, Subrata Banik, Maryanne M. Collinson

**Affiliations:** Department of Chemistry, Virginia Commonwealth University, Richmond, VA 23284-2006, USA; islamm3@vcu.edu (M.S.I.); baniks@vcu.edu (S.B.)

**Keywords:** nanoporous gold, bimetallic, electrochemistry, nanomaterials, nanoparticles, sensors

## Abstract

Bimetallic nanocomposites and nanoparticles have received tremendous interest recently because they often exhibit better properties than single-component materials. Improved electron transfer rates and the synergistic interactions between individual metals are two of the most beneficial attributes of these materials. In this review, we focus on bimetallic nanoporous gold (NPG) because of its importance in the field of electrochemical sensing coupled with the ease with which it can be made. NPG is a particularly important scaffold because of its unique properties, including biofouling resistance and ease of modification. In this review, several different methods to synthesize NPG, along with varying modification approaches are described. These include the use of ternary alloys, immersion–reduction (chemical, electrochemical, hybrid), co-electrodeposition–annealing, and under-potential deposition coupled with surface-limited redox replacement of NPG with different metal nanoparticles (e.g., Pt, Cu, Pd, Ni, Co, Fe, etc.). The review also describes the importance of fully characterizing these bimetallic nanocomposites and critically analyzing their structure, surface morphology, surface composition, and application in electrochemical sensing of chemical and biochemical species. The authors attempt to highlight the most recent and advanced techniques for designing non-enzymatic bimetallic electrochemical nanosensors. The review opens up a window for readers to obtain detailed knowledge about the formation and structure of bimetallic electrodes and their applications in electrochemical sensing.

## 1. Introduction

Nanoporous materials have had an enormous impact in many scientific and technological applications, including the development of adsorbents for cleaning up toxic waste, filtration and separation media for separating complex samples, catalytic materials for speeding up slow reactions, and chemical sensors for detecting trace constituents in complex samples, among others. Important properties include high surface area, a high surface area-to-volume ratio, high number of catalytically active sites, confinement effects, and unique and tailorable pore morphology. Examples of commonly used nanoporous materials include zeolites and clays, sol–gel-derived materials (xerogels and aerogels), the M41S family of materials, inverse opals, covalent organic frameworks (COFs), metal–organic frameworks (MOF), and nanoporous metals and foams [1,2,3,4,5,6,7,8,9,10]. The pore size, geometry, and interconnectivity strongly depend on the method of fabrication. Nanoporous metals are particularly valuable, because they have high electron conductivity, which allows them to serve as scaffolds for electrochemical devices such as electrochemical sensors, electrocatalysts, batteries, super capacitors, and fuel cells [11,12,13,14,15,16]. More importantly, they can have unique catalytic and electrochemical properties [13] due to their nanosized pores, high surface area-to-volume ratio, and large number of functional sites. The chemical and physical properties of nanoporous metals are often much better than those of nonporous metals, and thus they present exciting opportunities in the fields of chemical sensing, electrocatalysis, and energy storage and delivery.

One popular nanoporous material is nanoporous gold (NPG) [16,17,18,19,20,21,22,23]. NPG has been widely used in the field of analytical chemistry because of its unique pore structure and the ease with which it can be fabricated [18]. When prepared via dealloying methods, NPG has a characteristic three-dimensional bicontinuous nanopore framework, which has proven to be very valuable in electrochemical sensing [18] and electrocatalysis [19]. Compared to planar gold, NPG has many unique features [19]: (1) a high surface area that provides more places for electron transfer; (2) tunable pore size, ranging from a few nanometers to tens of nanometers, which allows pore-size-dependent effects to be evaluated [24,25]; (3) when prepared by dealloying in concentrated HNO_3_ [26], NPG has very clean surfaces, thus eliminating the need for harsh cleaning methods before use; (4) improved accessibility to internal sites due to the continuity and interconnectivity of pores [27]; and (5) high density of low-coordination surface gold atoms, which can improve electrocatalytic properties [28,29].

The unique properties of NPG have been exploited in several different ways. For example, NPG is a significantly better electrocatalyst for the oxidation of methanol than planar gold due to the presence of low coordination sites [30,31]. NPG is also effective for enzyme immobilization due to its small pores, which help stabilize the macromolecule under otherwise denaturing environments [32]. The high surface area of NPG means a greater amount of an adsorbate (e.g., enzyme) can be immobilized on the gold surface, allowing for greater signals relative to planar gold [24,33]. The bicontinuous, porous framework provides an avenue for small substrate molecules to reach inner surfaces and at the same time can reduce the effect of biofouling on an electrochemical signal due to a unique biosieving mechanism [34]. Because gold is easily modified with thiol groups, NPG can be modified with thiolated probe DNA molecules (or aptamers), enabling their use as DNA/RNA/nucleic acid sensors [35]. Electron transfer rates are also improved at nanoporous electrodes because of nanoconfinement effects [13].

While gold is an important element and has many useful attributes, it also has limitations. One important example is that gold is not as catalytically active as other metals. However, by either alloying gold or decorating gold with more catalytically active elements, improvements in the physicochemical properties of the nanoporous material can be obtained. Bimetallic nanocomposites and nanoparticles, in particular, have shown better physicochemical properties compared to their single-metal counterpart, and thus have shown their usefulness in chemical sensing and electrocatalysis [36,37,38,39,40,41,42,43,44,45,46,47,48,49,50,51]. Studies have shown that nanoporous alloys exhibit better electrocatalytic properties and have improved electrochemical sensing compared to single-component metals [37,52]. In some cases, these enhancements have been attributed to the synergistic interactions between the individual metals [37]. In chemical sensing, improved selectivity and sensitivity can also result from improved electron transfer rates when a second metal is added to a nanoporous structure [53,54]. Decorating a NPG framework with a precious metal can also reduce the consumption of that precious metal and provide high accessibility to it as needed.

In this review, we focus on the application of bimetallic NPG electrodes in electrochemical sensing. The reader is referred to reviews for the application of bimetallic, nanoporous composite materials that include NPG in electrocatalysis and fuel cells [37,49]. First, we briefly describe the major approaches for the fabrication of NPG including templating methods, chemical and electrochemical dealloying, and electrochemical methods such as anodization and surface roughing. We then discuss the integration of a secondary metal (Pt, Cu, Pb, etc.) into the NPG framework using different approaches such as immersion–reduction, electrochemical deposition–annealing, and underpotential deposition–redox replacement. This is followed by a description of the methods commonly used to characterize these materials, with an emphasis on the information that can be obtained about the physical and chemical structure of the bimetallic nanomaterial from each method. It is further emphasized in this section that the method of fabrication strongly influences the physical properties of the materials (pore size, pore morphology, surface area) and their chemical composition. Finally, applications of bimetallic NPG electrodes in the field of electrochemical sensing are then discussed, with particular attention given to the detection of glucose and hydrogen peroxide, among other analytes. By the end of this review, we hope the reader will better understand the synthesis, characterization, and applications of NPG and bimetallic NPG electrodes so that they can use these important materials to solve relevant chemical problems in the field of electrochemical sensing.

## 2. Fabrication

NPG can be made in many different ways. How NPG is made ultimately determines the pore structure, roughness factor, surface area, pore size, pore-size distribution, level of interconnectivity of the pores, and accessibility to the inner surfaces. All these properties are important, as they collectively determine the overall performance of the device or application. For electrochemical sensing applications, accessibility, surface area, pore size, and connectivity are especially critical, as they influence analytical figures-of-merit such as sensitivity, detection limit, response time, and selectivity. In this section, we briefly describe the most common ways of fabricating the NPG scaffold.

### 2.1. Templating

**Figure 1:**
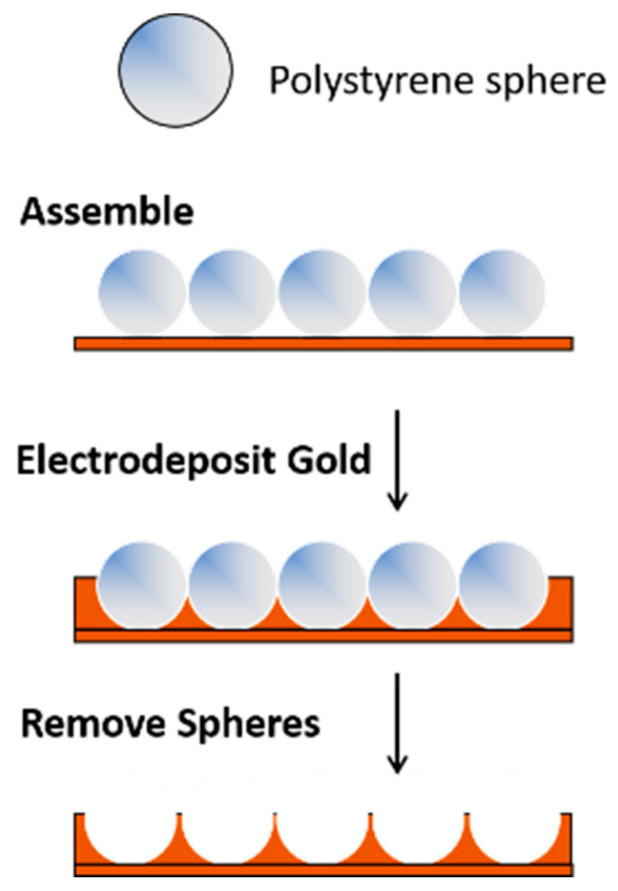
A templating approach used for the formation of porous gold electrodes.

Templating methods provide a simple approach for preparing porous gold electrodes with a wide range of pore sizes and/or unique nanostructures [3,4,8,55,56]. This approach involves the selection or fabrication of the template(s) and the formation of the metal (e.g., gold) framework around the template, which is then followed by template removal. For materials that require large pores in the 100+ nm range, hard templating methods are valuable. In this case, ‘hard’ sacrificial templates such as anodic aluminum oxide (AAO), large silica particles, colloidal crystals, or copolymer particles are used [8]. Gold (or another suitable metal) is electrochemically (or chemically) deposited in and around an assembly of hard templates, which is then subsequently removed by chemical means or via heat treatment [57,58]. Polystyrene templates can be easily removed in chloroform or toluene. An example of this approach is shown in Figure 1. The pores and the arrangement of the pores are dictated by the size of the template and how it is assembled on the surface. Because of the size of the templates (and ultimately the pores), the overall surface area of the electrodes is not as high as it is when smaller templates are used.

Soft templates can be used to produce NPG with significantly smaller pore sizes, often in the sub-10 nm range, thus increasing the surface area of the electrode [4]. Examples of such ‘soft’ templates include surfactant micelles, amphiphilic block copolymers, and lyotropic liquid crystals [55]. Bimodal porosity can be achieved by combining templates with different sizes and types [7] or by using hierarchical templates [59]. Hierarchical porosity can also be achieved by combining different soft templates or hard templates with soft templates (e.g., large polystyrene or silica particles with lyotropic liquid crystals) [7,60].

Many templates and approaches have been developed to create nanoporous (meso- and microporous) gold electrodes with different pore arrangements. For instance, a one-step liquid-phase method using AgCl as a sacrificial template was developed for preparing zero-dimensional hollow NPG with adjustable particle size (150–1000 nm) and ligament thickness (21–54 nm) [61]. Strawberry- and raspberry-like hierarchical templates [62] were fabricated by linking different-sized polystyrene latex spheres together, and they were used to prepare hierarchical nanostructured gold electrodes with large outer pores (~μm in size) and ~10 s of nm sized inner pores [59]. In another example, Hsueh et al. prepared NPG with a bicontinuous morphology by using different types of block copolymer (BCP) [63]. Using electrodeposited silica as a template, NPG with a coral-like framework and high surface area was fabricated [64]. The one drawback of template-based methods for NPG formation is that the overall structure of the nanoporous matrix is dictated by the shape of the template and template assembly. Different structures typically require the design of corresponding templates [34,59].

### 2.2. Chemical Dealloying of a Pre-Formed Alloy

One common method for preparing NPG with a bicontinuous network of nanosized pores is via chemical or electrochemical dealloying of a pre-formed alloy [27,65,66]. In this approach, the least noble metal in a gold alloy is selectively removed, ultimately producing a nanopore structure containing a bicontinuous, well-connected network of ligands and pores. Examples of Au alloys that have been used include Au-Sn [67,68], Au-Zn [69,70], Au-Cu-Pd-Ag-Si [71,72], Au-Ni [73], Au-Cu [74], Au-Fe [75], Au-Al [76], and Au-Ag [65,77], with the latter alloy being the most common. White gold (~12K; Au-Ag alloy) has been widely used to form NPG [77], in part because it can be purchased commercially in the form of thin white gold leaves (~100 nm thick). Such materials are commonly used by artists, thus making this material popular and readily available. An Au-Ag alloy of the appropriate composition could also be electrochemically deposited from appropriate gold and silver salts on a conducting support [78]. It can also be sputtered or physically deposited under vacuum using appropriate targets [79].

A simple method for chemically dealloying white gold is to place it in nitric acid for a selected period of time [34,80]. This time can range from ~10 min to hours, depending on the thickness of the starting alloy [18]. During this corrosion process, the least noble metal (silver) is depleted, and the gold atoms diffuse at the electrolyte–metal interface and restructure on the surface to make gold-rich clusters surrounded by nanosized pores [81]. The pore size and shape strongly depend on the concentration of the acid, the time in the acid, the composition of the alloy, etc. [18]. Chemical dealloying in acid is simple and quick, but the corrosion process, and thus the pore formation, can be difficult to control [37]. Additionally, it is impossible to remove all the Ag from the alloy, and care needs to be taken to fully understand what role residual Ag has on the observed response [82,83,84]. A recent approach has been described in which NPG is formed that is free from residual Ag [85].

### 2.3. Electrochemical Dealloying of a Pre-Formed Alloy

Another approach to dealloying that can provide more control is electrochemical dealloying [37]. In this approach, the material to be dealloyed serves as the working electrode and is immersed in a suitable electrolyte solution (e.g., perchloric acid) along with a reference and counter electrode. A potential slightly higher than the ‘critical’ potential is applied to the electrode surface with respect to a reference for a set period of time. The critical potential is very important, and its value depends on the alloy, its composition, and the electrolyte [86]. Linear-sweep voltammetry can help determine the critical potential [86]. For both chemical and electrochemical dealloying, post-processing procedures such as heat treatment and/or electrochemical treatment can be used to further tune the ligament and pore size.

### 2.4. Electrochemical Formation of the Alloy Followed by Electrochemical Dealloying

**Figure 2:**
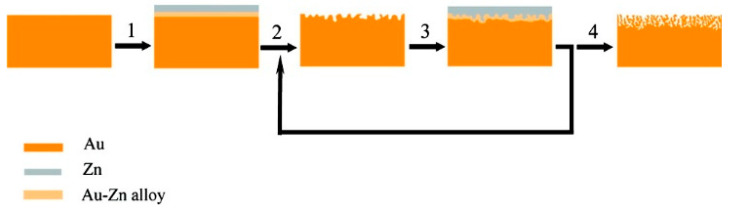
A simple representation of the formation of NPG via a multicycle electrochemical alloying–dealloying process. Reprinted with permission [69]. In step 1, Zn is electrodeposited and the Au-Zn alloy is formed; In step 2, the alloy is electrochemically dealloyed. The Au-Zn alloy is again formed in step 3 and high surface area gold is ultimately formed after this multicyclic alloying-dealloying process (step 4).

A ‘combined’ electrochemical alloying–dealloying process can also be used to prepare NPG [69,70]. In this method, instead of starting with a previously fabricated alloy, the alloy is made on the spot electrochemically and then immediately dealloyed in the same electrochemical cell. The process is a multicyclic process [69,70]. The method utilizes a ZnCl_2_/benzyl alcohol electrolyte solution held at an elevated temperature and a gold electrode. First, a cathodic sweep reduces zinc ions, forming an Au-Zn alloy, and second, the anodic sweep then subsequently removes Zn (i.e., causes dealloying). After several cycles, a 3D nanoporous structure is obtained [69]. A simple representation of the procedure is shown in Figure 2 [69]. This method is relatively simple, and can be used to form NPG layers on a gold wire or gold needles, which are very useful when small electrodes are needed for monitoring redox events in confined environments [87,88,89].

### 2.5. Anodization and Surface Roughening

**Figure 3:**
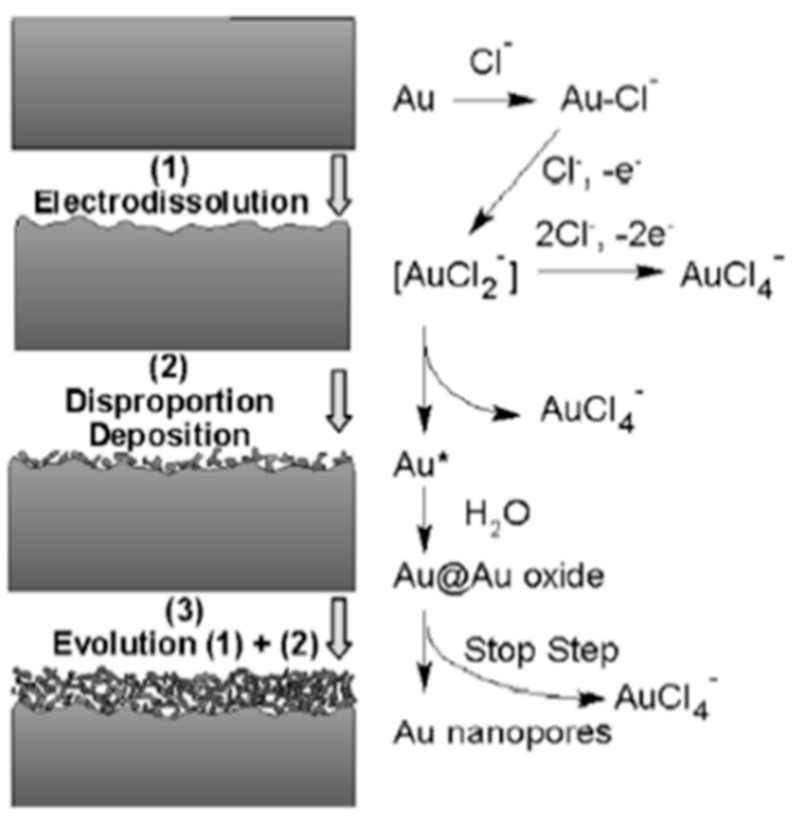
A simple representation of the formation of NPG via electrodissolution, disproportion, and deposition. Au* represents newly formed Au atoms. Reprinted with permission [94].

Another common and simple approach to prepare a high-surface-area NPG surface is anodization. This method is also conducive to the formation of NPG microelectrodes [90], and has commonly been performed using gold recordable compact disks cut into small pieces [91,92,93]. Different variants of this approach have been described in the literature. They typically involve, as a first step, the polishing or precleaning of the electrode surface. Next, the electrode is anodized via the application of a large positive potential in an electrolyte solution. In one example, 1.217 V (vs. Ag/AgCl) was applied to a precleaned gold microelectrode in HCl [90]. In another example, 0.9 V (vs. SMSE) was applied for 50 s [94]. An alternative procedure involves the application of 5 V (vs. Ag/AgCl) to a gold CD in phosphate buffer for three minutes [91,92,93,95]. Gas bubbles are produced, and an oxide is formed on the surface. The roughened electrode is then placed in a solution containing a chemical reducing agent (e.g., ascorbic acid) for a short period of time to reduce the gold oxide. Variations of this general approach include the application of square-wave potential pulses in sulfuric acid [96,97], the use of different electrolyte solutions such as phosphate buffer—KCl [98] or NaOH—with the use of a potential-pulse waveform from 1.2 to −4.6 V (vs. SCE) [99]. The mechanism for the formation of the roughened NPG surface depends on the reaction medium. In the case of KCl or HCl, the mechanism proceeds through electrodissolution, disproportion, and deposition [94,100]. A simple schematic of this process is shown in Figure 3. In NaOH, upon application of a square-wave potential pulse between 0.8 V to −5 V (vs. SMSE), NPG is formed via the weak release of oxygen gas followed by the intense release of hydrogen [99,101].

The advantages, disadvantages (concerns), and applicability of these methods are compared in Table 1.

## 3. Bimetallic NPG Electrode Fabrication

NPG is an important scaffold for applications in catalysis and chemical sensing, as it is easy to fabricate, has high density of low coordination sites, of coordination sites, a large surface area, a nanoporous structure, and unique chemical properties—particularly the ease with which gold can be modified [18]. However, NPG has limitations, and it is often necessary to modify its structure with other metals to improve its electrocatalytic and chemical sensing abilities [19]. For example, the addition of Pt results in an improved oxygen reduction reaction (ORR) and improved electron transfer kinetics for the detection of hydrogen peroxide. Because of synergistic effects, the resultant bimetallic material can exhibit better electrocatalytic abilities than either metal alone. Several different methods have been used to decorate or incorporate different metals into the NPG framework. These include the use of ternary alloys, immersion–reduction (chemical, electrochemical, hybrid), co-electrodeposition–annealing, and under-potential deposition (UPD) coupled with surface-limited redox replacement (SSLR). Examples of metals that have been incorporated into NPG using variations of these different approaches include Pt, Pd, Cu, Ni, Co, Ru, as well as their oxides, such as CuO, CeO_2_, MoO_2_, FeOOH, etc. In the sections that follow, these approaches to the formation of bimetallic and trimetallic NPG electrodes are described.

### 3.1. Ternary Alloys

One straightforward approach for the formation of bimetallic NPG electrodes is to start with a ternary alloy [102]. For example, NPG-Pt can be formed from an Ag-Au-Pt ternary alloy [102,103,104,105,106]. In one example, a master alloy composed of Au, Pt, and Ag was prepared via inductive or arc melting. Nanoporous Au-Pt was formed by electrochemically dealloying this master alloy in nitric acid [104]. In another example, Cu was removed from an Au-Pt-Cu ternary alloy (prepared by refining high-purity metal, followed by melt spinning) via electrochemical dealloying in sulfuric acid to form NPG-Pt with controllable composition [107]. More recently, Xie and Dimitrov prepared a nanoporous Au-Cu-Pt catalyst with low loadings of Pt by dealloying Cu from an Au-Cu-Pt alloy prepared via electrodeposition from a solution containing Cu, Au, and Pt salts [108]. Other examples include the dealloying of an Au-Pt-Al alloy (prepared by melting) in 4 M NaOH to remove the Al in order to prepare NPG-Pt [109], and electrochemical dealloying [110].

NPG-Pd nanostructures have also been prepared from ternary alloys. In one example, this bimetallic material was prepared using a one-pot approach from a chlorozincate ionic liquid via electrochemical alloying–dealloying of Zn on a PdAu substrate [111]. First, Zn was electrodeposited on the PdAu substrate to form a (PdAu)Zn surface alloy, before the anodic dealloying of Zn. In another example, a ternary Al-Pd-Au material was dealloyed in both single- and multistep processes in strong acids and/or bases in order to remove Al, resulting in a NPG-Pd nanocomposite [112]. While this approach is seemingly simple, a suitable ternary alloy of the appropriate composition is needed, but is often not readily available. Cost is another important factor to consider.

### 3.2. Immersion Followed by Reduction (Chemical, Electrochemical)

To add a secondary metal to a pre-formed NPG framework, perhaps the easiest and most versatile approach is to immerse an electrode (i.e., NPG) into a solution of a metallic salt and then electrochemically reduce the metal salt to the corresponding metal via the application of a controlled potential (potential step or cyclic scan) or a controlled current. This method works particularly well when the metal to be added to the NPG framework exists in a form (e.g., an aqueous salt) that can be easily reduced. Applied potential or applied current, electrodeposition time, and the concentration of the metal salt are important variables to control. Alternatively, the metallic salt can be chemically reduced by exposing it to an appropriate reducing agent. Both electrochemical and/or chemical reduction methods are relatively straightforward and have been used to create NPG–metal frameworks in which the metal is Pt, Cu, Pd, Ni, Co, or Fe. It is important in such experiments that the deposition of metal onto the framework does not block or clog the pores and restrict mass transport; it is also important to understand the location of the nanoparticles in the nanostructure (surface vs. bulk), the uniformity of deposition, and whether they are in a metallic state or in the form of an oxide. Proper chemical characterization is important.

**Platinum.** Platinum (Pt) is a particularly important metal because of its electrocatalytic properties. As a result, Pt has been the metal most widely used to modify NPG. Such bimetallic, high-surface-area materials are promising materials for the electrocatalytic oxidation of methanol, ethanol, and formic acid due to the synergy between the Pt and the Au in these alloys [30,113,114,115,116]. This can improve the efficiency of the ORR reaction [117]. Furthermore, Pt also exhibits good electrocatalytic activity towards hydrogen peroxide and glucose, thus helping to detect trace amounts of these analytes in chemical-sensing applications [117]. As noted in more detail below, the addition of Pt to NPG can further improve the chemical sensing of these and other important analytes. The loading of Pt into NPG can strongly influence activity.

**Figure 4:**
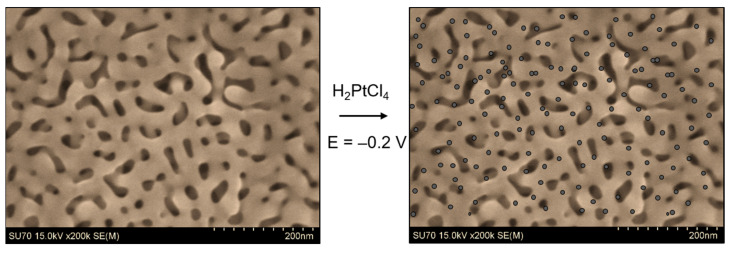
Simple representation of immersion followed by electrodeposition to prepare a bimetallic NPG. The procedure starts by immersing NPG (SEM image shown) into an acidic solution containing an appropriate metal salt (e.g., K_2_PtCl_4_), followed by electrodeposition. The gray circles represent electrodeposited Pt nanoparticles/islands.

One approach for incorporating Pt nanoparticles into the NPG framework involves immersing NPG in an acidic Pt salt solution such as H_2_PtCl_6_ or K_2_PtCl_4_ for a given period, and then exposing the metal-impregnated nanostructure to hydrazine vapor to form isolated Pt nanoclusters [30,118]. Instead of using a chemical reducing agent, the Pt ions incorporated into the nanoporous framework can be electrochemically reduced by applying a fixed potential or a cyclic scan. Several different approaches have been used. In one approach, the electrode was immersed in a Pt salt solution for a given period of time. The electrode was then removed from the solution and placed in an electrolyte solution, and the Pt salt present in the nanopores was electrochemically reduced [99]. In another case, the NPG electrode was placed in an acidic solution containing Pt salt, and the Pt ions in and around the electrode were subsequently reduced through the application of a suitable potential to that solution [54,80]. Of course, care must be taken so that the Pt does not fill the nanosized pores [80,119]. A simple depiction of the electrodeposition process is shown in Figure 4.

In a third approach, an atomic layer electrodeposition (ALED) procedure was used to control the growth of the Pt. First, Pt was electrochemically deposited, and then a capping layer of hydrogen was produced via underpotential deposition. This layer blocked the further deposition of platinum until a desorption potential was applied [98]. For the alteration of NPG with Pt layers, the authors showed that numerous cycles of ALED were more effective than a single cycle of ALED.

In a study by Y. Xue et al., a complex alloy was used. An Au_20_Cu_48_Ag_7_Pd_5_Si_20_ master alloy was prepared by arc-melting pure elements, before being dealloyed in nitric acid and HF at an elevated temperature [116]. Pt nanoparticles were then deposited electrochemically on NPG via cyclic voltammetry after immersing the electrode in H_2_PtCl_6_ in H_2_SO_4_ and cycling the electrode potential at 20 mV/s for 5 and 30 cycles. The Pt content after 30 cycles was 4.77 at %, and the particles were estimated to be 10s of nm [116]. In two other examples, Pt NPs were electrodeposited onto NPG using an electrolyte solution containing 0.5 M H_2_SO_4_ and 2 mM H_2_PtCl_6_ at a constant potential of −0.2 V (vs. saturated calomel electrode (SCE)) for 10 min [113] or electrolyte containing K_2_PtCl_4_ in 0.5 M H_2_SO_4_ at −0.2 V vs. Ag/AgCl [54,120].

S. Xiao et al. also reported a method for the deposition of Pt onto NPG by immersion of NPG in H_2_PtCl_6_ for a certain period of time in the dark at 25 °C, in order to avoid its decomposition by light. They obtained a Pt NP size of 3–5 nm, which was independent of immersion time, while the density of the nanoparticles on the surface increased with time [113]. Zhang and coauthors reported the modification of NPG by Pt by first immersing NPG in 96.5 mM H_2_PtCl_6_ for 10 min and then transferring the electrode to 1 M H_2_SO_4_ solution followed by sweeping the electrode potential. Low loadings (~7 at %) and good electrocatalytic properties were noted [114]. The authors argued that the good synergistic effect between Pt and Au played an important role in improving the catalytic activity of the electrode. In another approach, a NPG microelectrode was first modified with cysteamine hydrochloride and then immersed in a Pt salt solution for two hours. Electrodeposition proceeded upon application of −0.6 V (vs. Ag/AgCl) [87]. Modification of the electrode via the mercapto group was performed to improve the distribution and uniformity of the Pt nanoparticles [121].

**Other metals: Cu, Pd, Ni, and Co.** Copper/Copper (II) is another element that is easily oxidized/reduced upon application of a suitable potential. Copper nanoparticles/nanostructures improve glucose oxidation, and subsequently allow its non-enzymatic detection in different samples [122,123]. Similar approaches to that described for NPG-Pt can also be used to form Cu-NPG bimetallic nanostructures. Xiao et al. electrochemically deposited copper onto NPG using a fixed potential after immersing the NPG electrode in 10 mM CuCl_2_. The electrode was rinsed and placed in 0.1 M NaOH, and the potential was cycled to oxidize Cu to CuO and form NPG-CuO [122]. The authors reported a thickness of 5 nm for the CuO, which could be tuned by adjusting the deposition time and potential. They also reported on the effects of deposition time on glucose detection. In another example, Cu was electrodeposited onto NPG in 0.1 M CuSO_4_ under a constant cathodic current of 200 µA for 100 s (e.g., galvanostatic electrodeposition) [123]. Again, a very thin coating was formed (<2 nm thick). Bertotti et al. also recently reported the fabrication of Cu-NPG [124] by applying a constant voltage to NPG in 10 mM CuSO_4_ in H_2_SO_4_ for different lengths of time while stirring. In a more recent example, polypyrrole-Cu NPG electrode was made by co-reducing copper ions and pyrrole on a NPG electrode [95].

Examples of other metals that have been used to form NPG–metal frameworks for chemical sensing and electrocatalytic applications include Pd, Ni, and Co. The electrodeposition of palladium (Pd) onto NPG was carried out via cyclic voltammetry in a three-electrode electrochemical cell containing 5 mM SDS (sodium dodecyl sulfate) and 2.5 mM palladium chloride, with a potential window from 0.2 to 1.2 V vs. SCE [125]. A thin layer of Ni was electrodeposited onto NPG using a constant cathodic current of 200 µA for 100 s in 0.1 M Ni_2_SO_4_. Thickness was controlled by monitoring the total charge passed during the reduction process. Subsequently, NPG-Ni(OH)_2_ was produced by potential cycling from −0.8 to 0.14 V in 1 M NaOH. The thickness was estimated to be 4 nm [126].

**Figure 5:**
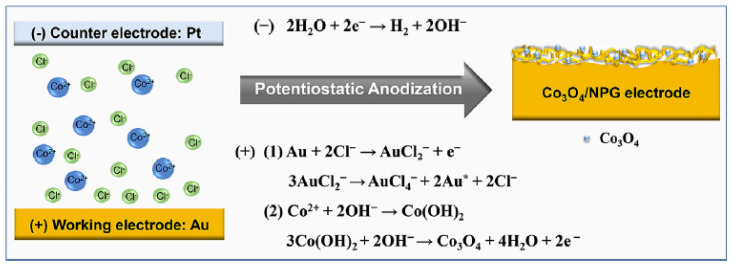
Schematic representation of a one-step approach for the formation of Co_3_O_4_-modified NPG. Reprinted with permission [128].

Regarding the deposition of Co or CoO_x_ onto NPG, Xia et al. reported a complex procedure that involved immersing a clean NPG electrode in a solution containing 3 mM Co(NO_3_)_2_, 3 mM EDTA, 3 mM K_3_Fe(CN)_6_ and 0.1 M KCl (pH 2.0), followed by continuous potential cycling. The last step involved transferring the electrode into 0.1 M NaOH followed by potential cycling to make CoO_x_/NPG [127]. In another example, a Co_3_O_4_/NPG electrode was produced in a one-step approach by immersing a gold electrode in CoCl_2_ solution and anodizing the electrode for 100 s [128]. A depiction of this one-pot method for the formation of a bimetallic electrode is shown in Figure 5 [128].

In addition to these examples, other metals and metal oxides have been incorporated into NPG. FeOOH nanoflakes were electrosynthesized in situ on a NPG microelectrode by applying 0.7 V (vs. Ag/AgCl, 1 M KCl) for 1800 s in a solution of sodium acetate containing 10 mM ferrous ammonium sulfate [89]. IrO_x_ was electrodeposited on NPG multielectrode arrays that were fabricated using sputter co-deposition of the Ag-Au alloy followed by patterning and dealloying. Cyclic voltammetry—up to 100 cycles—was used to deposit IrO_X_ as a thin layer on the gold surfaces [129].

In addition to electrochemical methods, a hydrothermal method has also been used to fabricate NPG-Co_3_O_4_ hybrid electrodes. First, the NPG gold electrode was produced via an electrochemical alloying–dealloying process; then, the electrode was immersed in a solution of cobalt nitrate and heated in a stainless-steel autoclave for 90 min. Both the concentration of Co(NO_3_)_2_ and the temperature were varied. The Co_3_O_4_ nanoparticles–nanocrystals with diameters of 5–20 nm grew into nanopores along the gold ligaments [130].

Two-step or co-electrodeposition approaches are also possible to incorporate multiple metals into the NPG framework. For example, Cui and coworkers described a two-step electrodeposition approach for fabricating a Pd@CeO_2_ NPG electrode on carbon fiber paper (CFP) [131]. The NPG-CFP was first immersed in a solution of Ce(NO_3_)_2_, and then the cerium ions were electrochemically reduced. Pd nanoparticles were then deposited by immersing the composite NPG electrode in a Pd salt solution, and the Pd ions were then electrochemically reduced. In another example, Guo and coauthor reported bimetallic Pt-Bi/NPG, where NPG-Pt was carried out by reducing H_2_PtCl_6_ using hydrazine hydrate vapor. Electrodeposition of Bi on the NPG-Pt surface was performed at 0.03 V (vs. SCE) in 5 mM Bi^3+^ and 0.1 M HClO_4_ [132]. A NiCo_2_O_4_-NPG electrode was also produced via co-electrodeposition at −0.8 V from a solution consisting of 5 mM Ni(NO_3_)_2_·6H_2_O and 10 mM Co(NO_3_)_2_·6H_2_O, followed by rinsing and then calcination at 300 °C in air for 2 h, thus forming NiCo_2_O_4_ nanosheets [88].

### 3.3. Electrodeposition–Annealing

A combination electrodeposition–annealing for the formation of a NPG–platinum bimetallic alloy was recently reported by Farghaly and coworkers [133]. In this approach, binary Pt-Ag alloys were electrodeposited from Pt and Ag salt solutions on a gold-coated slide and then annealed at 300 °C for 6 h. During heating, the gold atoms mixed with the Pt and Ag, forming a ternary alloy. Dealloying in nitric acid yields a nanoporous Pt-NPG framework. Different morphologies and differing compositions of Pt and Au in the nanoporous structure can be attained by changing the annealing time, temperature, and electrodeposition parameters [133]. In another case, Pt and Au salts were mixed in the appropriate mole ratio in sulfuric acid, and a very large voltage was applied (−3 to −5 V). The coalescence of the evolved hydrogen bubbles forms a Au-Pt nanoporous framework with large pores [134]. The advantage of these approaches is that the Pt and Au are mixed within and throughout the nanostructure, in contrast to when Pt is electrodeposited on pre-formed NPG. The addition of Pt can also help stabilize the NPG nanostructure [102].

### 3.4. UPD–Surface-Limited Redox Replacement

Another approach to modifying the surface of NPG with an ultra-thin layer of a metal or metal oxide is via under-potential deposition (UPD) coupled with surface-limited redox replacement (SSLR) [135]. UPD provides an avenue for modifying a metal surface at a nanometer scale and involves the deposition of a metal sublayer–monolayer onto the surface of a foreign metal at a potential that is more positive than the reversible Nernst potential [136]. Common UPD metals include Cu and Pb. When coupled with the SSLR reaction, it can be used to deposit a sub- to monolayer amount of the metal of interest onto a surface, including thin coatings of Ag, Cu, Pd, and Pt [135]. One advantage to applying this approach to NPG is that a very small amount of metal is deposited, allowing the nanopore framework to be maintained. Subsequently, the NPG framework can serve as a nanoporous template for fabricating other nanoporous metal electrodes. This approach has been used to introduce other metals such as Cu, Pt [137,138], Ru-Pd [92], Ru-Pt [139], Ni [140], and MoO_2_ [91] into the NPG framework when combined with SSLR.

In typical experiments, NPG is made using one of the methods previously described and followed by UPD of Cu or Pb on NPG. The Cu or Pb can then be replaced with another metal (e.g., Pt) as desired [137,141,142]. For example, an Au_1-x_Ag_x_ film was co-electrodeposited, and then the film was electrochemically dealloyed. The UPD of Pb on NPG followed by the SLRR of Pb with Pt yielded a NPG-Pt electrode with <1 nm thick Pt [143]. In another example, Au and Cu were co-electrodeposited to form a Cu_x_Au_1-x_ alloy. The alloy was then electrochemically dealloyed to form NPG. Pb UPD followed by SLRR with Pt yielded Pt-NPG [144,145]. In another example, a mesoporous Pt electrode was produced by starting with the NPG framework using a combination of the UPD of Pb followed by SLRR with Pt [90].

**Figure 6:**
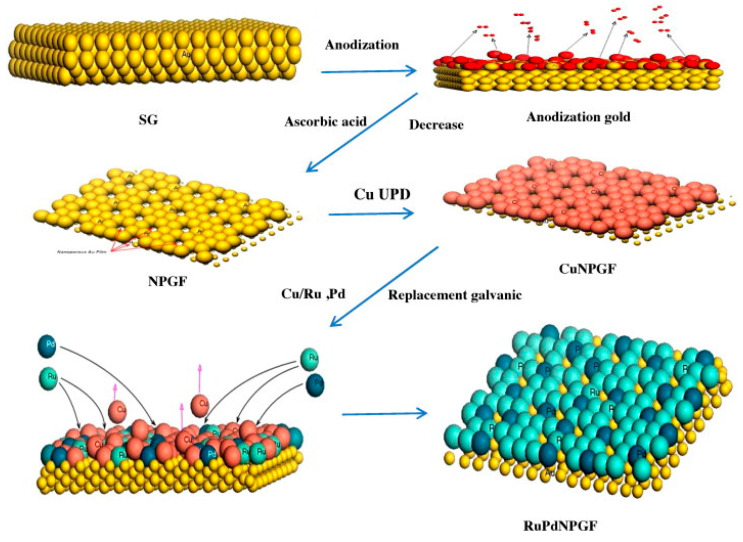
Schematic of the UPD approach for the fabrication of a trimetallic NPG. Reprinted with permission [92].

To introduce two metals into the system, as in the case of Ru-Pd, a NPG-Cu electrode formed via the UPD of Cu on NPG was immersed in an aqueous solution containing both RuCl_3_ and Pd(NO_3_)_2_ for the replacement reactions to take place [92,139]. A simple schematic of the UPD–SLRR process is shown in Figure 6 [92].

In another example, nanoporous Au-Cu foams were produced using hydrogen-evolution-assisted electrodeposition. Galvanic replacement of Cu by Pt leads to the formation of nanoporous ternary material (Pt@Au_x_Cu_100-x_), where the ratio of Pt to Au atoms can be controlled by adjusting the amount of Cu in the original foam [146].

Tavakkoli et al. reported the production of MoO_2_-modified NPG using the UPD-SLRR method, where a Cu UPD layer was formed on a gold electrode via application of 0.35 V vs. Ag/AgCl for 30 s in 1 mM CuSO_4_ in 0.1 M H_2_SO_4_. The subsequent replacement of Cu was carried out by immersing the Cu-NPG in 1 mM Na_2_MoO_4_ in 0.37 M H_2_SO_4_ for 10 min at an open circuit potential of 0.404 V [91]. The authors reported that a more uniform film could be formed using this approach than with direct electrochemical reduction.

**Figure 7:**
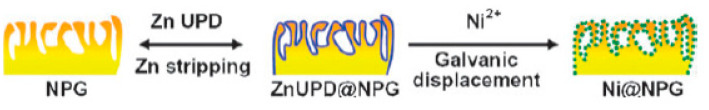
Preparation of a Ni-coated NPG electrode using galvanic replacement of Zn with nickel. Reprinted with permission [140].

In another example, Huang showed the preparation of Ni-NPG using Zn-NPG instead of using Cu-NPG, where Zn-NPG was made by scanning the electrode potential from 0.5 to −1.0 V, before holding it at −1.0 V (vs. SCE) for 3 min; then, the ZnUPD@NPG was immersed in 50 mM NiSO_4_ at open circuit for 3 min to obtain Ni-NPG (Figure 7) [140].

The advantages, limitations (concerns), and applicability of these methods for the formation of bimetallic NPG are compared in Table 2.

## 4. Characterization

Morphology, surface area, porosity, pore distribution, and elemental composition are very important properties of a high-surface-area support as they will influence the overall performance of the material. Pore size and pore continuity will impact the diffusion of a reactant into and products out of a nanoporous framework, which is critically important in catalysis and chemical sensing. They can also impact the uniformity with which a secondary metal can be incorporated into the NPG framework, and thus influence the figures-of-merit (for chemical sensing) or catalytic efficiency. In the case of NPG prepared by dealloying, the percentage of silver (or another metal) remaining in the material after dealloying is important. Even small amounts can influence the results. For example, NPG prepared from Au-Ag alloy by dealloying in concentrated nitric acid contains ~92% Au and ~8% Ag [80]. When NPG is decorated with another metal, the location of the metal, the size of the metal, its distribution, and the oxidation states need to be considered. It is important to know whether changes in pore size or accessibility occur upon modification, as these attributes can affect material response. Many techniques have been used to investigate the physical and chemical characteristics of NPG and bimetallic NPG. The most common ones include Scanning Electron Microscopy (SEM), Transmission Electron Microscopy (TEM), X-ray diffraction (XRD), and electrochemical techniques. X-ray Photoelectron Spectroscopy (XPS) or Auger Spectroscopy and Scanning Electron Microscopy–Energy-Dispersive X-ray spectroscopy (SEM-EDX) have been used to characterize the elemental composition. These methods and the types of information that can be obtained from them are described below.

### 4.1. Scanning Electron Microscopy (SEM)

**Figure 8:**
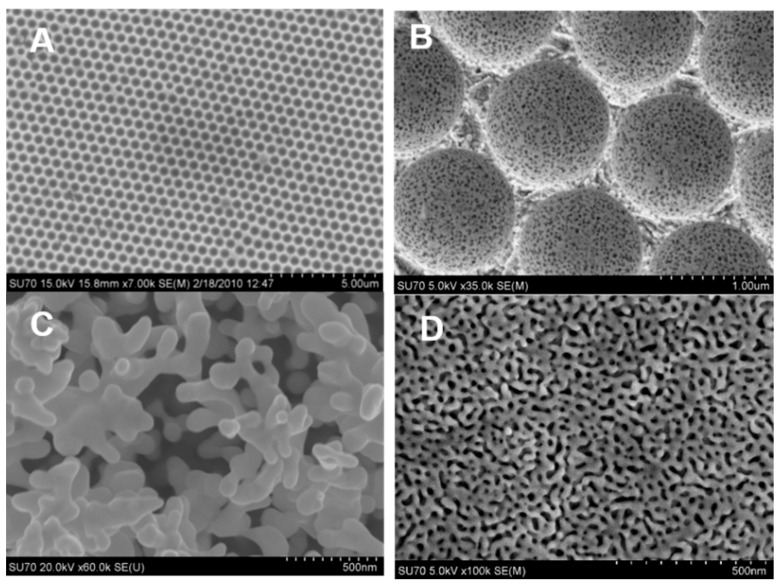
SEM images of nanostructured gold electrodes prepared by (**A**) templating with latex spheres, (**B**) templating with raspberry latex spheres, (**C**) using silica as a sacrificial alloy, and (**D**) dealloying white gold leaf in concentrated nitric acid.

SEM is one of the most popular methods for characterizing the surface morphology of NPG and bimetallic NPG. NPG’s inherent conductivity eliminates the requirement for a thin metal coating on the samples before imaging can begin, resulting in sharper results. However, it is important to point out that SEM only captures a relatively small area of the total surface. The presence and degree of microscopic cracking in the sample can be revealed in low-magnification images. High-resolution SEM images can provide valuable information about pore size, while cross-sectional SEM images can provide information about film thickness and pore connectivity. The average pore size and ligament size of the pores in NPG, for example, can be estimated using image analysis software [147]. Figure 8 presents SEM images obtained from nanostructured gold electrodes made in different ways. It is most evident from these SEM images that the microstructure of the material is highly dependent on how it is made. In Figure 8A,B, the nanostructured gold was made via templating methods using a hard template (polystyrene latex spheres). Figure 8C shows NPG produced using electrodeposited silica as the template, while Figure 8D presents an SEM of NPG prepared by chemically dealloying white gold leaf in nitric acid. Distinct pores (holes) can be seen in all of the structures except that prepared from electrodeposited silica, which is more representative of a roughened electrode.

**Figure 9:**
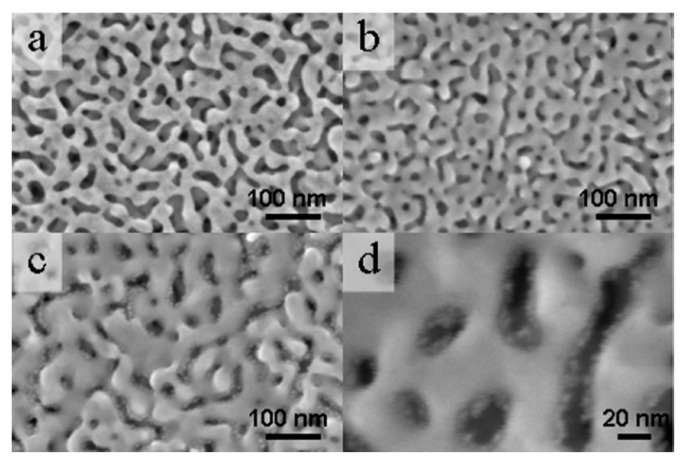
SEM images of (**a**) NPG and (**b**,**c**) NPG-Pt at different plating times (2 and 128 min). (**d**) High-magnification SEM showing the Pt particles in the nanopores when heavily plated. Reprinted with permission from [30].

SEM is also a valuable tool for evaluating the microstructure of bimetallic materials. It is an easy tool to use for determining whether the addition of a second metal to a nanoporous framework, such as that deposited electrochemically or chemically via the reduction of a salt, has altered the morphology of the electrode. In high-resolution images, it is possible to see the small nanoparticles/clusters on the surface of the nanoporous framework. A specific example is shown in Figure 9, where NPG is decorated with Pt using the immersion method followed by chemical reduction of the Pt salt. When NPG is heavily modified, small Pt clusters can be seen, with an estimated size of 3 nm (Figure 9d) [30].

**Figure 10:**
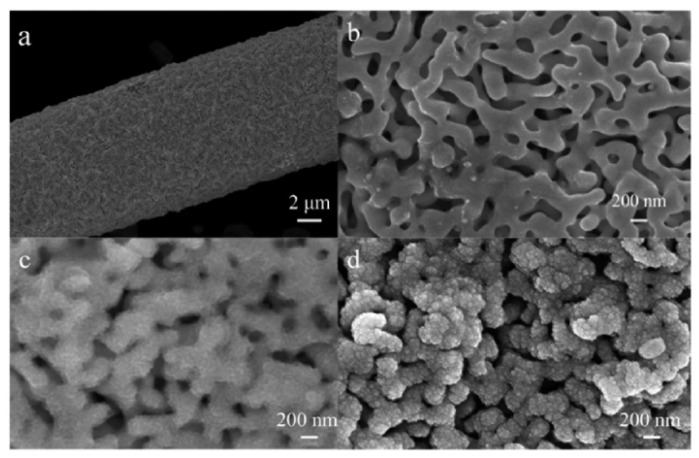
(**a**) SEM images of NPG formed on a carbon fiber by the electrodeposition of Au-Sn alloy followed by electrochemical dealloying. (**b**) High-magnification SEM image showing the NPG film. (**c**,**d**) High-magnification SEM images of the NPG film after the addition of CeO_2_ (**c**) and, subsequently, Pd (**d**). The nanoparticles appear evenly distributed. Reprinted with permission [131].

SEM is also a straightforward tool for visualizing and following the addition of different metals to the nanoporous gold surface. Figure 10 shows the SEM images of a Pd@CeO_2_ NPG at various points during its fabrication. In this work, Cui and coworkers first formed NPG coated on carbon fiber paper (CFP); Figure 10a,b show the SEM images [131]. Next, the NPG-CFP was immersed in a solution of Ce(NO_3_)_2_ and the cerium ions were electrochemically reduced. Pd nanoparticles were then deposited by immersing the composite NPG electrode in a Pd salt solution, and the Pd ions were then electrochemically reduced. SEM images confirm confirm that the NPG film uniformly covers the carbon fiber surface. High magnification reveals the morphology of the NPG, Figure 10b. The surface became rougher when CeO_2_ was deposited, but the porous structure was still present (Figure 10c). It can be seen from Figure 10d that the Pd nanoparticles are uniformly dispersed on the CeO_2_ layer [131].

### 4.2. SEM-Energy Dispersive X-ray Spectroscopy (EDX)

When SEM is coupled with EDX, the elemental composition of the material can also be determined. This information can be highly valuable. It can, for example, report how much of the least noble metal is left in the framework after chemical or electrochemical dealloying. For NPG prepared by dealloying gold leaf, SEM-EDX is routinely used to evaluate the %Ag that remains after dealloying. When an additional element is added, SEM-EDX can be used to estimate how much of that element has been incorporated into the material. This is particularly useful when experimental variables are changed.

**Figure 11:**
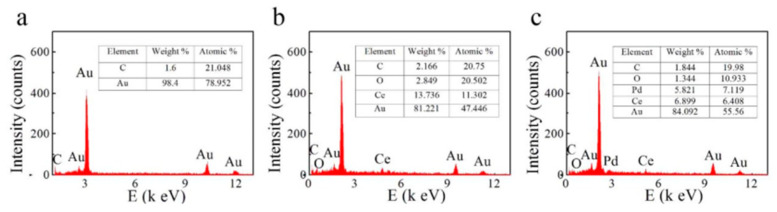
EDX spectra acquired on (**a**) NPG/CFP, (**b**) CeO_2_/NPG/CFP and (**c**) Pd@CeO_2_/NPG/CFP. Reprinted with permission [131].

Using Pd@CeO_2_ NPG as an example, EDX proved to be very useful for determining how much Ce and Pd were incorporated into NPG, evluating whether all the Sn had been removed during electrochemical dealloying of the Au-Sn alloy, and also confirming the presence of CeO_2_ from the atomic ratios of O to Ce [131]. Figure 11 shows EDX images for the different materials made in this work. Figure 11a confirms the removal of Sn from the Au-Sn alloy used to make NPG. Figure 11b,c confirm the addition of both Ce and Pd, which were electrochemically deposited on the nanostructure [131]. EDX elemental mapping also proved to be valuable in this work, because the authors were able to use it to evaluate the uniformity of the deposited metal on NPG from elemental maps of Pd, Ce, Au, and O.

### 4.3. X-ray Photoelectron Spectroscopy (XPS)

**Figure 12:**
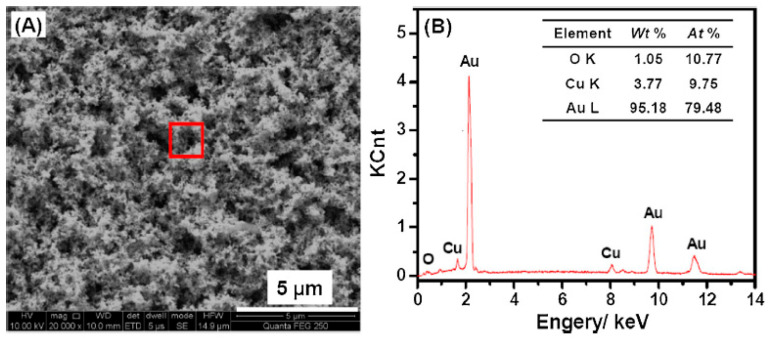
SEM image and corresponding EDX of a Cu-NPG electrode. Reprinted with permission [123]. (**A**) SEM; (**B**) EDX spectrum. The red box in (**A**) is the location where the EDX spectrum was collected.

**Figure 13:**
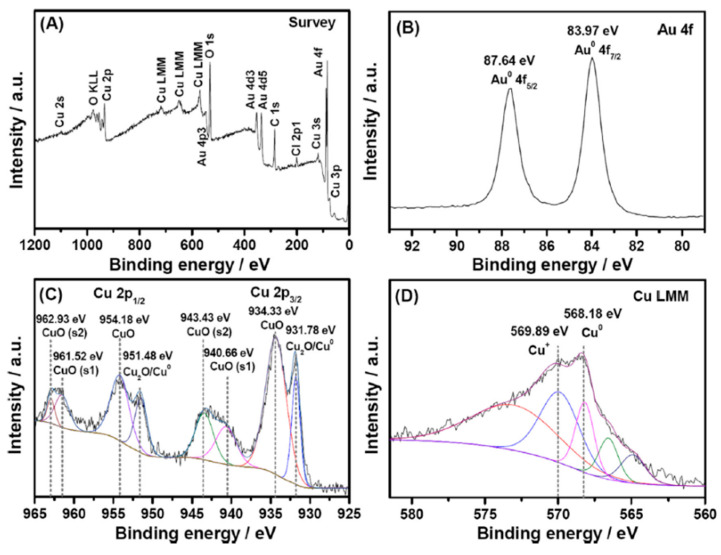
XPS spectra of a Cu-NPG electrode with fittings appropriate for evaluating the oxidation state of copper in the electrode. Reprinted with permission [123]. (**A**) Survey scan; (**B**) Au 4f spectrum; (**C**) Cu 2p spectrum; (**D**) Cu LMM spectrum. The different colors indicate the fits to the experimental data.

Another valuable method for quantitatively determining the elemental composition of high-surface-area supports is XPS. XPS is more sensitive than SEM-EDX, it offers greater spectral resolution, readily determines the oxidation states of metals, and integrates only the top few nanometers of a surface, unlike EDX. Care must be taken that carbon contamination does not influence the results obtained. XPS results can be compared to those obtained using SEM-EDX; collectively, these two measurements can provide elemental information on the surface vs. the bulk, respectively. Metals have distinct XPS spectra, and the binding energy depends on the oxidation state. One example of the power of XPS and EDS was noted in the work undertaken by Guo and coworkers, who formed a Cu-NPG electrode [123]. A NPG electrode was made by anodizing a gold electrode in KCl, followed by immersing it in HCl to remove the surface oxide layer. Cu was electrodeposited from a CuSO_4_ via a galvanostatic electrodeposition process. Figure 12 shows the SEM image and the corresponding EDX result. The SEM in Figure 12A shows the morphology expected for a roughed gold electrode and the EDX in Figure 12B provides an initial idea of the composition of Au and Cu in the material [123]. The Cu coating is very thin, as the pore network can still be observed in the SEM image. XPS was used to confirm the oxidation state of the Cu. Figure 13A presents the XPS spectra for the survey, Figure 13B presents the XPS spectra for gold and Figure 13C,D present the XPS spectra for copper [123]. The characteristic doublet of Au can be seen, and the different oxidation states of Cu can also be noted.

**Figure 14:**
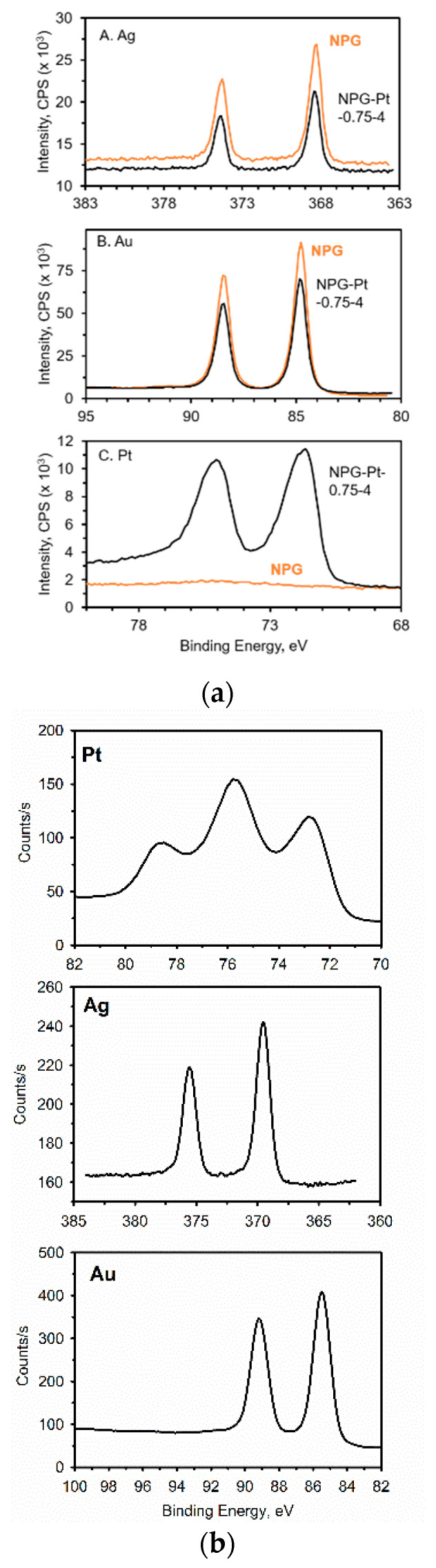
XPS spectra obtained on NPG-Pt electrodes. (**a**) The electrode was produced by electrodecorating a dealloyed gold leaf with Pt nanoparticles/clusters [80]. (**b**) The electrode was prepared by dealloying a ternary Pt-Ag-Au electrode [133]. The Pt is in very different oxidation states in the two different materials.

XPS has also been used as an excellent tool for evaluting bimetallic NPG-Pt materials, examples of which are shown in Figure 14. In Figure 14a, the XPS results obtained from a NPG-Pt electrode are shown. The NPG was prepared by dealloying gold leaf in nitric acid, followed by immersion in a Pt salt solution and application of a sufficiently negative potential to reduce the Pt ions to small Pt nanoparticles/clusters [80]. Before the electrodeposition of Pt, the characteristic doublets of Au and Ag can be seen. Following electrodeposition, the characteristic Pt doublet appears. All three elements show characteristic doublets. The %Au, %Pt and %Ag can be readily determined from such spectra by taking into account the sensitivity factors [80]. Very different results were observed for a NPG-Pt composite electrode prepared in a different fashion, Figure 14b. In this study, a Pt-Ag film was formed on a gold electrode via co-electrodeposition of a platinum and silver salt solution followed by heat treatment. During annealing, Au atoms diffuse into the Pt-Ag layer to form a ternary alloy [133]. Upon dealloying in nitric acid, Ag is removed, and a NPG-Pt electrode is produced. The characteristic doublets for Au and Ag can be seen, but the Pt XPS shows a more complicated spectrum consisting of Pt oxides with different oxidation states [133].

### 4.4. Transmission Electron Microscopy (TEM)

**Figure 15:**
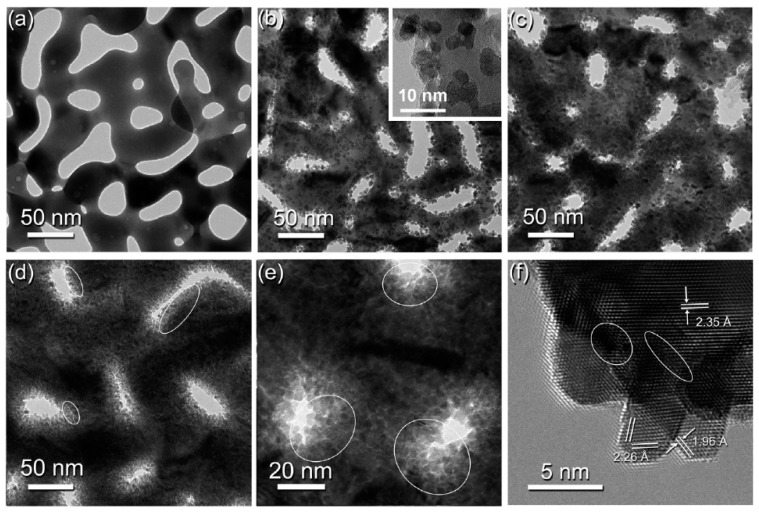
TEM images of NPG before (**a**) and after Pt deposition for different lengths of time: (**b**) 0.5 h, (**c**) 1 h, and (**d**,**e**) 3 h. A high-resolution image is shown in (**f**) depicting Pt anchored to the gold skeleton. Reprinted with permission [113]. The white ellipses shown in (**d**–**f**) signify the nanoscale interstitials between the Pt nanoparticles.

As a quantitative approach for evaluating particle size, form, and distribution, transmission electron microscopy (TEM) is a favorable tool. Since TEM allows the direct capture of high-resolution images, it is one of the best methods for analyzing the morphological characteristics of nanomaterials [148]. TEM offers higher spatial precision and the potential to perform analytical measurements, making it a more attractive option than SEM under some circumstances. However, the samples must be thin and transparent. When compared to SEM, TEM provides atomic and crystallographic data by creating 2D images that are simpler to understand than 3D SEM images, and permit users to investigate more characteristics of a given sample. Moreover, TEM is ideal for studying NPG decorated with nanoparticles or thin films [23]. In addition to elemental data, thinly prepared samples can reveal the dimension of the nanoparticles scattered across the NPG’s surface [87,149]. An example is shown in Figure 15, which shows NPG decorated with Pt nanoparticles formed after deposition for different lengths of time. A high-resolution image clearly shows the Pt nanoparticles (Pt-NP) anchored to the walls of the NPG. With increasing deposition time, Pt-NPs become denser. An increase in the deposition time from 0.5 to 3 h causes a change from monolayer to multilayer Pt-NP stacking, as is evident from the TEM images shown in Figure 15b–d. The size of the Pt-NPs is ~3.5 nm, and appears to be independent of the deposition time. Based on the lattice spacing (Figure 15f), the epitaxial growth of pure Pt-NPs instead of Pt-Au alloy is confirmed [113].

### 4.5. XRD

**Figure 16:**
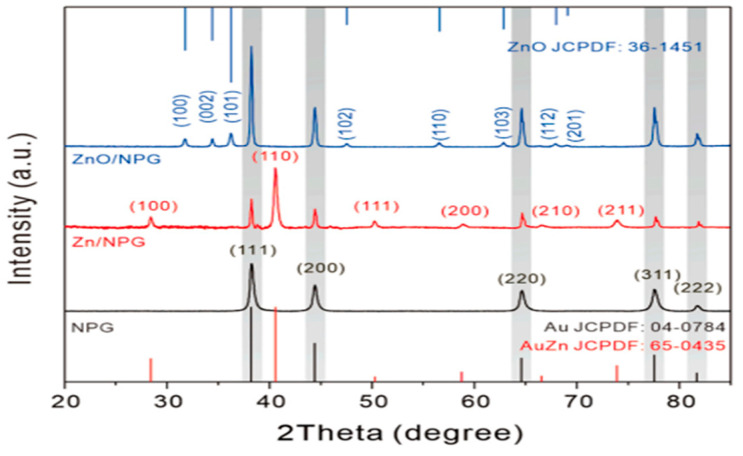
XRD patterns: NPG (black line), ZnO/NPG (blue), and ZnNPG (red). Reprinted with permission [150]. The shaded parts indicate strong peaks for FCC Au.

There are many methods for characterizing nanoparticles, but X-ray diffraction (XRD) is among the most common. XRD is a common technique for determining a crystal’s structure, phase, lattice parameters, and particle size. While the previous techniques provided ways of characterizing the morphology of nanoparticles, XRD is good for understanding the crystal size distribution and the nature of phases. By analyzing the sharpness of the diffraction peak, the crystallinity of a solid can be obtained. Through this, it can be determined whether a solid particle is a single or poly crystal. Using the Scherrer equation, the average size of the nanoparticles dispersed on the NPG can also be obtained. An example of XRD data is shown in Figure 16 for a NPG electrode modified with ZnO via thermal treatment [150]. Peaks for face centered cubic (FCC) Au, Cubic AuZn, and hexagonal ZnO were discovered when XRD was used to examine the crystalline structure of the in situ-formed Zn nanostructure on the NPG surface and the subsequent thermally treated product (Figure 16). The ZnO crystallite size on the surface of the NPG was calculated to be approximately 29.1 nm using the Debye–Scherrer equation [150].

### 4.6. Surface Area Measurements

Many different approaches can be used to measure the real surface area of electrode materials, and these include gas sorption methods such as N_2_ Brunauer, Emmett, and Teller (BET), and electrochemical methods such as UPD, electrochemical impedance spectroscopy, and cyclic voltammetry (CV), among others [151,152]. The BET technique is useful when a large amount of material is present, as in the case of nanoporous foams, aerogels, or monoliths. It does not work well with films, due to limited amounts of material present on the electrode surface, or in cases where the material is temperature sensitive and undergoes chemical and physical changes, such as coarsening during outgassing. This is especially problematic for NPG [137]. Electrochemical methods are particularly useful and effective for the measurement of ‘real’ or electrochemically active surface area of conducting materials such as nanoporous electrodes [151]. In the case of nanoporous electrodes, the surface area can be tens to hundreds of times larger than the geometric area due to the presence of the nanosized pores. By dividing the electrode’s effective surface area by its geometric equivalent, its roughness factor (RF) can be determined. Table 3 presents some examples of RFs obtained for NPG electrodes made using the methods described in a previous section. It is obvious from this table that the surface area depends on the electrode fabrication method.

As can be seen, roughness factors can range in value from ~3 to over 100, depending on the method of fabrication. In general, NPG made using anodization methods gives rise to larger areas than those prepared by via hard templating with latex spheres. Precise knowledge of the real surface area is very important in the field of electrocatalysis and chemical sensing, as high areas can yield greater numbers of chemically active sites [157]. Several different methods can be used to measure the surface area of nanoporous materials [147,157]. In this work, we focus on electrochemical methods [158], most notably, cyclic voltammetry and underpotential deposition (UPD). Each has its advantages and disadvantages [147,151,157].

**Figure 17:**
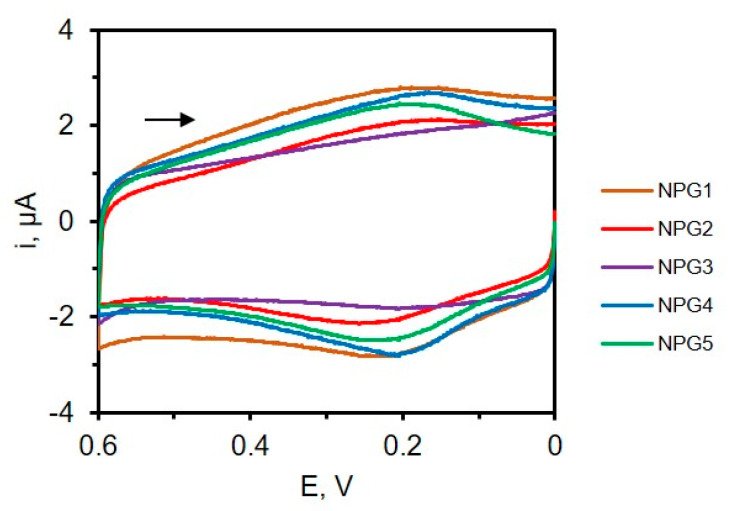
Cyclic voltammograms of NPG electrodes (N = 6) in 0.1 M KCl at a scan rate of 50 mV/s. The arrow signifies the direction of the potential scan.

We typically start by acquiring cyclic voltammograms (CV) in the double-layer region, where no Faradic processes take place. This approach can be used to evaluate the reproducibility of electrode fabrication. Three or four electrodes can be made, placed in an electrolyte solution, and CVs undertaken at a specified scan rate using a multichannel potentiostat. By overlapping the CVs for the different electrodes, it is possible to visually see variations in the active surface area of the electrodes by focusing on the width of the CV. Figure 17 shows an example of CVs acquired using six different NPG electrodes prepared by dealloying in concentrated HNO_3_ for 40 min. The RSD in the capacitive current obtained at 0.5 V (vs. Ag/AgCl) is ~10%. This approach for qualitatively evaluating reproducibility in electrode fabrication is non-destructive and the electrode can often be used in other experiments. Quantitative estimation of the area, however, requires knowledge of the capacitance of the material, which is often not available.

**Figure 18:**
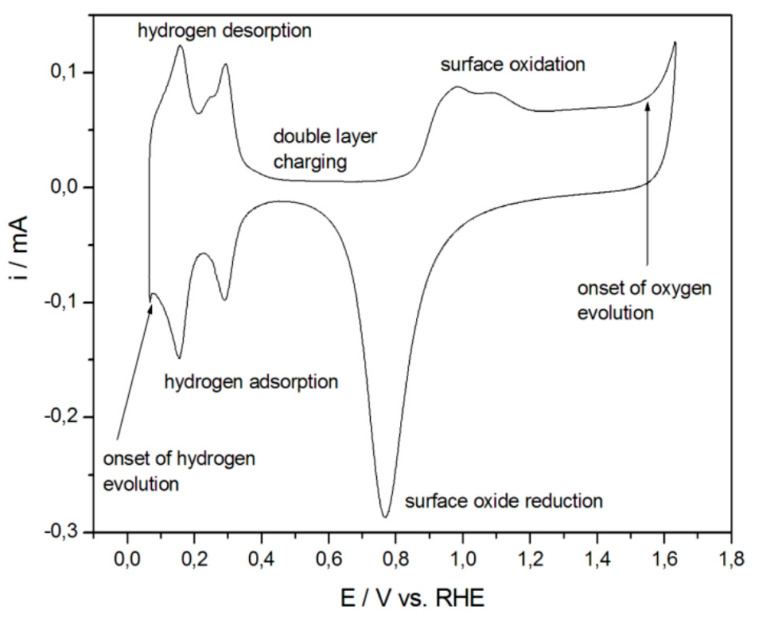
CV acquired at a Pt electrode in 0.5 M H_2_SO_4_ at 0.1 V/s. The hydrogen adsorption and desorption areas are used to estimate the real surface area of the electrode, as shown. Reprinted with permission [158].

For Pt, quantitative measurements of the real surface area are typically undertaken by placing the electrode in a solution of sulfuric acid and scanning the electrode potential over the hydrogen adsorption–desorption region. By carefully integrating the voltammetric peaks in the desorption region and by dividing the measured charge by the charge due to the oxidation of a monolayer of the adsorbed hydrogen per unit area (e.g., 210 μC/cm^2^), the real surface area of the electrode can be found. Figure 18 shows a CV acquired at a Pt electrode that depicts the hydrogen desorption–hydrogen evolution region [158].

**Figure 19:**
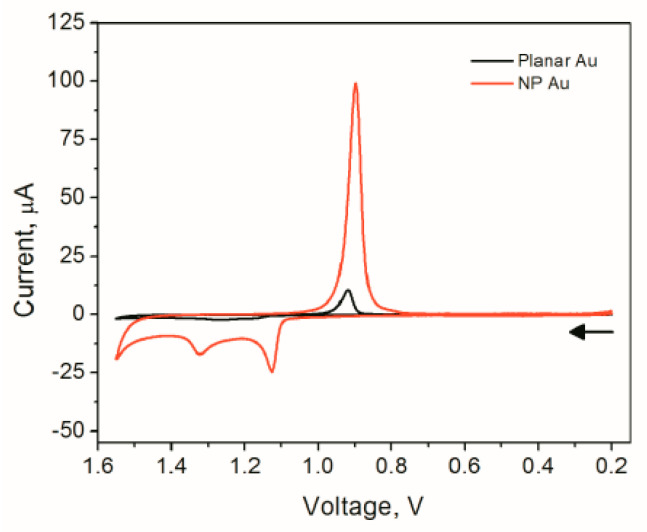
CVs acquired using planar gold and NPG electrodes in 0.5 M H_2_SO_4_ acquired at 0.1 V/s. The arrow signifies the direction of the potential scan.

For Au, the surface oxide reduction approach is typically used to measure the electrochemically active surface area. In this experiment, the electrode is placed in sulfuric acid, and the electrode potential is scanned over an appropriate region to oxidize the gold and then reduce the gold oxide that was formed. The charge under peak is measured and divided by the charge density due to the reduction of one monolayer (386 μC/cm^2^) to obtain the real surface area of the electrode [158]. A similar approach can be used for Pd [157]. The charge density associated with the reduction of one monolayer of PdO is 424 μC/cm^2^ [157]. Figure 19 shows the CVs acquired for NPG obtained by dealloying white gold leaf. The peak at ~0.9 V represents the reduction of the gold oxide, and is used to measure the real surface area of the electrode. It can be easily noticed that the real surface area of NPG (red line) is significantly higher than that obtained using a planar gold electrode (black line) of the same geometric area.

**Figure 20:**
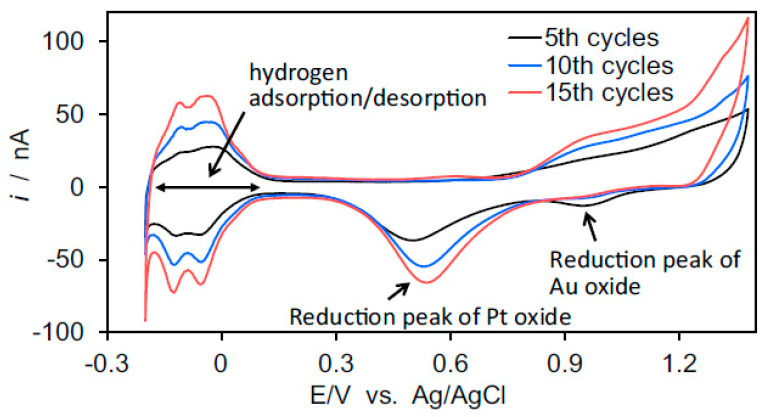
CVs acquired using a NPG microelectrode coated with a thin Pt film using UPD of Pb followed by SLRR of Pt for different numbers of cycles. CVs were acquired in 0.5 M H_2_SO_4_ at 100 mV/s [90].

For alloys composed of both Au and Pt in sufficient concentrations, such as bimetallic Pt-NPG, it is possible to use a single CV to estimate the area of both Pt and Au. It is also possible to estimate how much Pt covers the gold electrode by examining the decrease in charge under the gold oxide peak as the gold is covered by Pt [80]. An example is shown in Figure 20 [90]. In this example, a high-surface-area NPG electrode is produced via anodization. A thick film of platinum was grown on the skeleton of NPG via multiple UPDs of lead (Pb) followed by SLRR cycles. It can be noted from this figure that as the Pt is incorporated into the NPG with increasing numbers of the UPD-SLRR cycles, the hydrogen adsorption–desorption peaks increase in amplitude, as does the Pt oxide peak, while the gold oxide reduction peak decreases.

Two other common electrochemical approaches for measuring the real surface area of the electrode are UPD and electrochemical impedance spectroscopy (EIS). The UPD of Pb and Cu on NPG has been used to measure the real surface area of the electrode [159]. However, for nanoporous electrodes prepared by dealloying that have residual metal in them (e.g., Ag for NPG prepared by Au-Ag alloy), this method could underestimate the real area. This is because Cu UPD, for example, is not active on Ag [147]. Due to its reliance on the model and fitting techniques employed, EIS can be a little challenging. A comparison of electrode surface areas obtained using the surface oxide reduction method, the UPD method, and the impedance method for nanoporous gold electrodes prepared via dealloying has been reported [92].

## 5. Electrochemical Applications in Chemical Sensing Using Bimetallic NPG Electrodes

Chemical sensors are powerful analytical tools that are used to detect and quantify the amount of an analyte in a sample. They have been used in a wide range of applications including clinical, environmental, industrial, agricultural, and diagnostic testing to detect different molecules through a catalytic or binding event that occurs at the electrode interface or through electron transfer events [160,161,162,163,164]. Electrochemical sensors are very important because they are simple to use, construct, portable, economical, and do not require expensive instrumentation [165]. One very important part of an electrochemical sensor is the electrode, with gold, platinum, glassy carbon, and some semiconductor electrodes being most common due to their inertness and good electrochemical properties. Electrodes play an important role because they facilitate electron transfer, influence surface adsorption, and help detect the target molecules. High-surface-area materials, ideally with an interconnected nanopore network, are needed to amplify the electrochemical signal, to load more of the biorecognition element onto the surface (e.g., protein or enzyme), and to increase the electron transfer rates for electrochemically slow redox reactions [165,166].

Electrochemical sensors can be classified into enzymatic and non-enzymatic sensors based on the presence or absence of enzymes [166,167]. Of the two types of electrochemical sensors, non-enzymatic sensors have come into the limelight and the enzymes have been replaced with low-cost, solid inorganic nanocatalysts [165,168]. Non-enzymatic sensors are much more convenient, due to their simplicity of preparation and operation, good stability, high sensitivity, and low cost compared to enzymatic sensors [165]. However, it has been well established that electrode materials play a critical role in the construction of these high-performance electrochemical sensing platforms for detecting target molecules.

Considerable efforts have been devoted to incorporating different metals and metal oxides into high-surface-area porous frameworks to improve the electrochemical properties of the surface, most notably, rates of electron transfer [37]. The decoration/modification of nanoporous gold electrodes with other metals is no exception, and these materials have been proven to be valuable in chemical sensing applications, as described in more detail in the following sections. The best aspects of the NPG framework and its associated high surface area, coupled with the electrocatalytic properties of the added metal/metal oxide, can make for effective electrochemical sensors.

### 5.1. Hydrogen Peroxide (H_2_O_2_) Sensing

Hydrogen peroxide is a particularly important molecule, because it plays a vital role in biological processes such as oxidative stress [169], in addition to being a molecule widely used in the chemical and food industries [167,170]. Different methods have been reported for the detection and measurement of hydrogen peroxide, including electrochemistry, fluorometry, titrations, spectrophotometry, and chemiluminescence [171]. Electrochemical methods utilizing enzymatic and nonenzymatic electrochemical sensors stand out because of their low cost, simplicity, high sensitivity, and/or wide linear detection range [170,172]. Of these two approaches, non-enzymatic chemical sensors for hydrogen peroxide are the most promising because of their lower cost, greater stability, and longer lifetimes. However, weaknesses include the need for higher overpotentials due to slow electron transfer and poisoning of the electrode surface [168]. Bimetallic nanoporous materials, in particular, can help to reduce or mitigate these limitations by increasing the surface area of the electrode, improving ET rates and thus reducing overpotentials, and in some cases reducing the effects of biofouling on the electrochemical signal [34].

While the ET kinetics for the reduction of H_2_O_2_ are much better with NPG than with planar gold [173], the modification of NPG with Pt can further improve performance. For example, Chong Xiao et al. [87] fabricated NPG microelectrodes and decorated them with Pt to create a composite electrode for sensitively reducing H_2_O_2_. These electrodes were also used for monitoring H_2_O_2_ release from a human breast cancer cell. Amperometric monitoring was carried out for MCF-7 cells, with phorbol myristate (PMA) being used to generate H_2_O_2_ in cells. In a more recent example, Pt-decorated NPG was fabricated and used to measure H_2_O_2_ from PC12 cells stimulated with ascorbic acid [119].

Potentiometric detection of H_2_O_2_ has also been achieved using a Pt-decorated NPG electrode, with good sensitivity [54]. Unlike voltammetric or amperometric methods, potentiometry is a net-zero current measurement and does not appreciably alter solution concentrations. It involves measuring the open-circuit potential difference between the sensing electrode and a stable reference electrode using a high-impedance voltmeter. The measured potential varies logarithmically with concentration, and only those molecules that can efficiently transfer electrons with the electrode surface are detected. Islam et al. showed that the decoration of NPG with Pt nanoparticles/islands significantly improved the potentiometric sensitivity of the electrode toward hydrogen peroxide [54]. A more than 3-fold increase in sensitivity was achieved when a Nafion coating was used in conjunction with Pt-decorated NPG [54].

In another example, Khan and coauthors [174] reported a nanoporous Pt-Au composite electrode that was able to detect hydrogen peroxide in the presence of albumin (under biofouling conditions). The composite electrode was fabricated via electrodeposition of a Pt-Ag alloy on a gold electrode followed by annealing at a high temperature and dealloying. The NP-Pt (Au) electrode showed faster kinetics for H_2_O_2_ reduction compared to either planar Pt or NPG. The overpotential needed to reduce H_2_O_2_ was significantly lower on bimetallic NPG electrode, as evidenced by cyclic voltammetry. Even in the presence of albumin, a common biofouling agent, the electrode could still detect H_2_O_2_ with minimal reduction in signal. The detection of H_2_O_2_ under biofouling conditions is possible because of the unique pore network of the nanoporous bimetallic electrode [174].

Apart from Pt-NPG for H_2_O_2_ biosensing, other approaches have been employed. Ke et al. described a three-dimensional NPG/Ni foam hybrid electrode for the electrochemical reduction of H_2_O_2_ [175,176]. In this case, an Au-Sn alloy was electrodeposited on Ni foam and subsequently dealloyed. The NPG improved the stability of the Ni foam in acidic solution and the electrode was able to detect H_2_O_2_ in acidic media with improved durability [176]. A Co_3_O_4/_NPG electrode was also used to detect H_2_O_2_, and demonstrated good sensitivity under alkaline conditions. In this case, a one-pot strategy was used to produce the composite electrode [128]. In another example, a free-standing NPG was fabricated and decorated with CoO using the ALD method and used to detect H_2_O_2_ with good selectivity and sensitivity [177]. Table 4 summarizes some figures-of-merit for bimetallic NPG electrodes recently used for the electrochemical detection of H_2_O_2_.

### 5.2. Glucose Sensing

Glucose is an equally important molecule in biological systems and the need to quantitatively assess its concentration in clinical samples is also of paramount importance. Again, both enzymatic and non-enzymatic electrochemical approaches have been used [179,180,181,182]. The direct oxidation (or reduction) of glucose is particularly promising because it does not require an immobilized enzyme (e.g., glucose oxidase) to function. While such biosensors show good selectivity and sensitivity, long-term stability is often limited. While the direct electrochemistry of glucose eliminates the need for an immobilized enzyme, it is not without its challenges. Slow electron transfer rates, electrode poisoning from oxidation intermediates, and reduced selectivity can be particularly problematic [179,181]. Nanoporous metals, such as bimetallic and other composite materials, have been particularly beneficial and have provided new opportunities for glucose sensing [182]. The synergy between the different metals can result in an improved electrocatalytic response [179,180,181]. A few examples of NPG-based bimetallic electrodes for the direct detection of glucose are described herein.

Noble metals such as Pt and Au and their alloys have shown considerable promise for the fabrication of non-enzymatic amperometric glucose sensors. In one example, a NPG electrode fabricated by a square-wave potential-pulse treatment in sodium hydroxide was decorated with Pt nanoparticles via immersion in a Pt salt solution followed by electrodeposition. This electrode was used to oxidize glucose under acidic, basic, and neutral conditions. The amount of Pt incorporated into the NPG framework strongly influenced the electrocatalytic activity [99].

Other transition metals, such as Ni, Cu, Co and their alloys, have also been very useful in direct glucose sensing. In one example, a NPG-Ni electrode was fabricated in a multistep process. First, NPG was prepared by dealloying Zn from a AuZn alloy [140]. Next, the surface was modified with a Zn-UPD adlayer followed by galvanic displacement with nickel. The detection of glucose was undertaken in an alkaline solution with excellent sensitivity [140]. NPG-Ni(OH)_2_ was prepared by electroplating Ni onto the surface of NPG. XPS confirmed that the electrode was composed of Ni(OH)_2_, Ni, and Au. The electrode was used to detect glucose in basic conditions. Compared to NPG, the NPG-Ni(OH)_2_ electrode showed enhanced electrocatalytic activity; the catalytic rate constant was 34X greater than that on NPG. A Cu-coated NPG electrode also showed good results for the detection of glucose [123]. In this case, the NPG was made by an anodic potential step approach and then a thin layer of copper (~1.7 nm via TEM) was coated onto the surface using electrodeposition. The NPG had a very high roughness factor. The authors state that this bimetallic electrode had excellent electrocatalytic activity for the oxidation of glucose due to the nanoporous structure and the ‘pronounced co-mediating’ of CuII/CuIII and Au/AuI [123]. Bertotti and co-authors [124] also fabricated a bimetallic Cu-NPG electrode via a two-step process and used it to detect glucose. First, NPG was made by electrodepositing gold onto a screen-printed electrode followed by electrodeposition of Cu from an acidic solution of CuSO_4_. These electrodes were used to detect glucose in salvia samples. Compared to NPG, the Cu-NPG electrode showed better results in terms of peak potential and peak current density regarding the anodic oxidation of glucose [124].

Bimetallic nanoparticles have also been used in conjunction with a NPG framework. In one example, Zhao et al. [183] prepared PtCo alloy nanoparticle-decorated NPG-supported graphene paper. This flexible composite electrode was made by capturing a dealloyed gold leaf on free-standing graphene oxide paper and held via Nafion [183]. The PtCo was deposited electrochemically from a solution containing cobalt and nickel salts; XPS confirmed the formation of the alloy, which also contained a small amount of metal oxide. The composite flexible electrode showed better electrocatalytic activity for glucose oxidation compared to NPG–graphene under basic conditions. They attributed this improved performance in part to high surface area and a synergistic catalytic effect [183].

In another example, NPG needles were prepared using an electrochemical alloying–dealloying process and then decorated via electrodeposition from a solution containing both a nickel and cobalt salt. After annealing at 300 °C, a NPG-NiCo_2_O_4_ electrode was formed [88]. Improved performance for glucose sensing in alkaline media over NPG was noted and attributed to the hierarchical nanostructure and exposure to more active sites [88]. Additionally, Li et al. [184] fabricated a two-dimensional (2D) bimetallic NiCo metal–organic framework (MOF) electrode on NPG using a bottom-up approach without surfactant. In this case, the NPG served as the scaffold to direct the vertical growth of the Ni-Co-MOFs. The electrode showed excellent behavior in terms of mass and charge transport of the electrochemical reactions with more accessible sites. Glucose was detected under basic conditions, as well as in human blood serum. High sensitivity and low detection limits were obtained. The authors attribute such promising results to the ‘synergistic coupling’ effect of N, Co, and Ni in the NiCo-MOFs [184]. Additional examples and a comparison of some analytical figures-of-merit between the different electrodes are shown in Table 5.

### 5.3. Sensing Applications of Bimetallic Decorated NPG for Molecules or Ions Other Than H_2_O_2_ and Glucose

Bimetallic NPG electrodes have been used in chemical sensing applications for a variety of different analytes in addition to hydrogen peroxide and glucose. Examples include protons (e.g., pH), small biomolecules and antioxidants, medication, organic molecules, and metal ions. Some of these applications are described herein. More details and figures-of-merit can be found in Table 6.

Pt-decorated NPG electrodes have been used in the potentiometric sensing of ascorbic acid, cysteine, uric acid, dopamine, and associated mixtures. Unlike traditional amperometric measurements, open-circuit potential (OCP) and redox potential measurements are not selective toward a particular species and are strongly influenced by the rates of electron transfer, which can be altered by decorating an electrode with nanoparticles [53]. In work by Islam et al., NPG was prepared by dealloying white gold leaf; Pt nanoparticles/islands were then electrodeposited on the NPG electrode from solutions containing a Pt salt. The OCP was measured at different concentrations. The incorporation of Pt significantly improved the sensitivity of the potentiometric measurement [80], as did the use of Nafion [54]. Sensitivities of −118 mV decade^−1^ change in concentration were obtained for dopamine by using Nafion-coated Pt-decorated NPG. Furthermore, Nafion imparts selectivity to the surface for the detection of mixtures containing low concentrations of dopamine and ascorbic acid [54].

Recently, Pt-decorated NPG was used for the electrocatalytic oxidation of small organic molecules (SOMs) with applications related to technologies such as fuel cells, pesticides, and pharmaceutical synthesis along with the clinical diagnosis of cancer. [115,185,186]. The as-reported bimetallic electrode showed enhanced catalytic activity, and a low limit of detection, and durability. X. Ge and coauthors, for example, reported the use of Pt-decorated NPG for the electrocatalytic oxidation of formic acid. They prepared bimetallic Pt-NPG by reducing H_2_PtCl_6_ onto NPG by the vapor of hydrazine hydrate followed by dealloying in concentrated HNO_3_; these electrodes showed double catalytic activity of bare NPG [115]. Y. Pei et al. reported the synthesis of binder-free Pt-decorated NPG (Pt-NPG) by electrodeposition of Pt from H_2_PtCl_6_ onto highly roughened NPG formed via the anodization of AuSn alloy in HCl [185]. The authors showed that the electrode had excellent catalytic activity towards the electrooxidation of hydrazine, good selectivity, and a high sensitivity compared to bare NPG [185]. Methods other than chemical etching have also been used to prepare NPG. A three-dimensional Pt-NPG was prepared by electrodeposition from a Pt-precursor, H_2_PtCl_6_, onto NPG prepared by square wave voltammetry in H_2_SO_4_ solution. The as-synthesized electrode showed better sensing towards miRNA detection with amplified signal and anti-interference capability than NPG [186].

Pt-decorated NPG has also been used to fabricate pH-sensitive electrodes. The NPG essentially serves as a scaffold for Pt/Pt oxides, which forms an equilibrium with H^+^ [187]. Euna Lee and coauthors [98] reported on Pt-modified NPG electrodes for pH sensing. The NPG electrodes, prepared by anodization of gold, were coated with Pt via atomic layer electrodeposition for various time frames. The Pt-covered NPG electrode exhibited enhanced pH response compared to that obtained on uncoated NPG [98].

N. Tavakkoli et al. synthesized bimetallic monolayer (e.g., Ru-Pd and Ru-Pt) onto NPG for electrocatalytic oxidation of captopril and methionine, respectively [92,139]. They prepared NPG through the anodization of a smooth gold surface in 0.1 M phosphate buffer solution at 5 V (vs. Ag/AgCl) and subsequent reduction by ascorbic acid to convert gold oxide to metallic gold in both cases. They decorated bimetallic nanoparticles by co-deposition of the respective ions (Ru and Pd and Ru and Pt ions) on NPG by oxidation of a Cu UPD layer. The observed results showed that galvanic replacement provides a simple, facile method for fabricating bimetallic nanoparticle-decorated nanoporous-gold-film electrodes with better catalytic efficiency and selectivity compared to NPG.

Bimetallic NPG electrodes have shown considerable promise in the detection of mercury and arsenic. Mercury (Hg) and arsenic (As) are recognized as global pollutants, as they pose serious threats to human health and the environment. Therefore, it is very important to detect these pollutants with simple and cost-effective methods. For example, A. Chen et al. [89] reported FeOOH-decorated NPG prepared via electrodeposition at 0.7 V (vs. Ag/AgCl) from sodium acetate precursor, while the NPG was prepared by electrochemical alloying–dealloying using CV in ZnCl_2_ solution from −0.7 to +1.8 V using a zinc foil counter and zinc wire reference electrode. Excellent electrochemical performance was achieved with a high sensitivity of 123.5 μA μM^−1^ cm^−2^ and a low detection limit of 7.81 nM. In addition, no obvious interference from common ions (e.g., Cu(II), Pb(II), Cd(II)) was observed, and the FeOOH/NPG microelectrode demonstrated exceptional stability. The microelectrode showed good stability over 30 days, with an RSD of 2.63% [89].

Z. Zhuang et al. [150] fabricated a ZnO/NPG electrode by thermal treatment in air. The NPG electrode was prepared via the electrochemical alloying–dealloying method and used in the electrochemical analysis of As(III) in real samples (tap and lake water) with a satisfactory recovery rate of 91.4% to 101.3% (10 ppb) and 88.1–97.6% (100 ppb), respectively. A high sensitivity of 1.366 μA⋅ppb^−1^·cm^−2^ with a low limit of detection of 0.30 ppb (S/N = 3) was achieved at concentrations ranging from 1.0 ppb to 260 ppb [150]. The microelectrode showed good electrochemical stability and no significant changes in current density at 60 ppb and 100 ppb As(III), with RSD values of 4.42% and 2.68%, respectively.

G. Li et al. [131] fabricated Pd@CeO_2_-decorated NPG-coated carbon fiber paper (CFP) for the electrochemical determination of 4-aminophenol. CFP was electrodeposited onto NPG, where NPG was prepared by applying a constant potential of 0.7 V (vs. Ag/AgCl) in an Au-Sn precursor solution. Then, CeO_2_ was deposited via CV from 10 mM Ce (NO_3_)_3_ using a potential window of −0.2 V to −1.2 V. Finally, Pd particles were deposited onto CeO_2_/NPG/CFP by chronoamperometry at −0.6 V vs. Ag/AgCl electrode. The catalytic activity of the as-prepared electrode toward 4-aminophenol was significantly increased due to the presence of Pd/CeO_2_ particles and higher surface area and conductivity resulting from the hierarchical porous structure [131]. It manifested an extremely high sensitivity of 75.4 mA mM^−1^ and 56.5 mA mM^−1^ in concentration ranges from 0.005 mM to 0.03 mM and 0.03 mM to 9 mM, respectively.

N. Tavakkoli and coauthors [91] reported on a molybdenum (IV)-oxide-decorated NPG (MoO_2_) for the detection of methimazole. The electrode was prepared by UPD of Cu on NPG and then the spontaneous replacement of the Cu layer by a thin layer of MoO_2_. The NPG was prepared by anodizing the smooth gold surface in 0.1 M phosphate buffer at 5 V and the subsequent reduction of gold oxide to metallic gold by ascorbic acid. The observed electrode showed high sensitivity toward methimazole and lower anodic overpotential [91].

Sadeghi et al. [95] reported polypyrrole/CuO nanocomposite-modified NPG for the electrochemical determination of piroxicam and tramadole. Electrochemical deposition of CuO nanoparticles and electro-polymerization of pyrrole were subsequently carried out onto NPG after preparing NPG by anodization in phosphate buffer via OCP at %V and reduction with ascorbic acid. The fabricated electrode showed long-term stability and better sensing performance toward the mixture of piroxicam and tramadole than NPG [95].

In other work, titanium-chitosan-decorated NPG for the electrochemical sensing of acetaminophen in the presence of piroxicam has been described [188]. Titanium was decorated onto NPG by the UPD method at 0.9 V vs. (Ag/AgCl) and chitosan (CS) was deposited onto Ti/NPGF at a constant potential of 3 V. The prepared electrode showed better sensitivity toward acetaminophen in the presence of piroxicam, but was ineffective in the presence of uric acid, glucose, vitamin E, and ascorbic acid [188].

**Table 6:** Examples of metal and metal-oxide-decorated NPG electrodes for sensing ions and organic molecules and relevant figures-of-merit.

Electrode	Method	Analyte	Conditions	Linear Range	LOD, nM	Sensitivity	Interference Study	Ref.
NPG-Ti-Chitosan	DPV	acetaminophen	Buffer, pH 7	60–700 μM	10		Yes	[188]
ZnO-NPG	SWASV	As(III)	0.1 M PBS, pH 5.0	1.0–260 ppb	0.30 ppb	1.366 µA ppb^−1^cm^−2^	Yes	[150]
NPG/ITO	DPASV	As(III)	0.1 M HCl	0.1–50 µg/L	0.054 µg/L	9.837 μA μg L^−1^	Yes	[189]
FeOOH-NPG	SWV	Hg(II)	0.1 M PBS; pH 5.0	0.02–2.2 µM	7.81	123.5 μA μM^−1^ cm^−2^	Yes	[89]
np-Au NPs/ITO	DPASV	Hg(II)	0.1 M HCl	0.1–10 µg/L	0.15		Yes	[190]
Pt-NPG	CA	Hydrazine	0.2 M PBS, pH 7.0	5 μM to 6.105 mM	1030	3449.68 μA mM^−1^ cm^−2^	Yes	[185]
Pd@CeO_2_-NPG/CFP	CA	4-aminophenol	0.1 M PBS; pH 7.0	0.005–0.03; 0.03–9 µM	4	75.4 and 56.5 µA µM^−1^	Yes	[131]
MoO_2_/Cu-NPG	DPV	methimazole	0.1 M PBS; pH 7.0	0.01–30 µM	35	4.3 μA μM^−1^	Yes	[91]
RuPt-NPG	DPV	methionine	0.1 M PBS; pH 7.0	0.006–0.105 and 3–102 μM	2	0.063 μA μM^−1^	Yes	[139]
RuPd-NPG	CA	Captopril	0.1 M PBS; pH 7.0	0.0025–0.475 and 2.5–32.5 µM	1.25	0.022 mA μM^−1^	Yes	[92]
PPY-CuO-NPG	DPV, CV	Piroxicam and tramadole	0.1 M PBS; pH 7.0	0.05–30.0 & 50.0–300.0 µM	10	0.428 μA μM^−1^	Yes	[95]
Pd-NPG	DPV	Dopamine	PBS	1–220 μM	1000	1.19 μA μΜ^−1^	Yes	[125]

SWASV—square-wave anode-stripping voltammetry; DPASV—differential-pulse anode-stripping voltammetry; HNPG—highly roughened nanoporous gold; SWV—square wave voltammetry; CV—cyclic voltammetry; CFP—carbon fiber paper; CA—chronoamperometry; PPY—polypyrrole.

## 6. Summary and Future Directions

Bimetallic nanoporous gold (NPG) electrodes play an important role in the field of chemical sensing. These electrodes exhibit the promising properties of NPG—such as high surface area, tunable pore structure, and ease of fabrication—along with electrocatalytically active sites achieved by the addition of metallic or bimetallic metals to the nanoporous framework. The net result is an electrode with improved sensitivity and performance. Often, very little of the element (e.g., Pt, Cu, etc.) is needed, making this approach a cost-effective means of fabricating electrochemical sensors without the incorporation of enzymes. Control over the amount of the added metal or metal oxide to the NPG electrode provides an avenue to tune performance.

In this review, we show how relatively easy it is to modify the composition and structure of bimetallic NPG electrodes to match the needs of a variety of different applications whether it be for the detection of hydrogen peroxide, glucose, or small neurotransmitters. We also show how important it is to properly characterize the materials both in terms of the amount and location of the added metal, but also its impact on pore morphology and connectivity. It is also evident from this review that the sensitivity, detection limit, and stability can be improved by adding other metals, metal oxides or metal alloys to NPG, which can then be used to detect a wider variety of analytes and enhance the sensor’s overall performance. The sensing capabilities of bimetallic NPG composites have been expanded through the integration of functional components like metal oxides. By combining the exceptional qualities of NPG with the targeted capabilities of the other added metals, these composites provide synergistic benefits that improve sensing performance, selectivity, and stability.

The diverse range of chemical sensing studies emphasizes the broader applicability and versatility of bimetallic NPG electrodes in addressing critical sensing challenges. As we look toward the future, we expect to see continued exploration in the design and fabrication of bimetallic NPG electrodes, as well as their applications in the field of chemical sensing, particularly for analytes that would otherwise exhibit slow rates of electron transfer at NPG alone. Redox potentiometry, in particular, is very highly dependent on the rates of electron transfer and it is expected that bimetallic NPG electrodes will increase the applicability of this method in the field of chemical sensing. The fabrication of bimetallic or trimetallic NPG in which the individual metals are fully integrated into the electrode, in contrast to just being decorated onto the outer surfaces, offers many additional avenues for synergistic interactions leading to improved response. One-step approaches for making these fully integrated bimetallic nanoporous electrodes would be beneficial. At a more basic level, a more thorough understanding of the fundamental electrochemical processes occurring at the electrode surface would also be beneficial to the field. Finally, more applications of such electrodes for analyte detection in real-world samples with complex matrices, in particular, will further show the promise of these interesting materials.

## Figures and Tables

**Table 1:** Summary of Common Fabrication Methods for Porous Gold Electrodes.

Method	Requirements	Advantages	Concerns
Templating Hard Soft Hierarchical	Template(s) needed. The template must maintain its structure during fabrication and be able to be completely removed.	Wide range of pore sizes (nanometer to micron). Can be used to fabricate hierarchical NPG electrodes.	Requires a template of a suitable size and shape. Cleanliness of the electrode surface upon removal of the template. Lower roughness factors for hard templating.
Chemical dealloying a pre-formed alloy	Alloy of suitable composition containing the desired metal.	Chemical dealloying is very easy, does not require a potentiostat, and the surface is typically very clean. Adaptable to many different alloys.	Chemical dealloying does not provide a lot of control over the dealloying process.
Electrochemical dealloying of a pre-formed alloy	Alloy of suitable composition containing the desired metal.	Electrochemical dealloying provides control over the dealloying process, particularly compared to chemical dealloying. Dealloying produces a bicontinuous network of pores.	Requires the use of a potentiostat and the need to know the critical potential.
Electrochemical alloying–dealloying	Zn-Au alloy.	Higher surface areas are possible. Easier adaptivity to the formation of nanoporous microelectrodes.	Requires the use of a potentiostat. Limited in its adaptivity to other metals beyond gold.
Anodization–Roughening	Metal that can be roughened electrochemically.	Simple and can provide high surface area. Easier adaptivity to the formation of nanoporous microelectrodes.	Bicontinuous network of pores is not present. Limited in scope compared to other methods.

**Table 2:** Summary of common fabrication methods for the formation of bimetallic NPG.

Method	Requirements	Advantages	Concerns/Limitations
Ternary alloys	A suitable ternary alloy of the appropriate composition.	Straightforward.	Requires a ternary alloy of a suitable composition.
Immersion followed by reduction (chemical, electrochemical)	Reducible metal salt.	Simplicity and versatility. Can be used to simultaneously add multiple different metals to NPG. Adaptable to many different metals (Pt, Cu, Pd, Ni, etc.). Chemical reduction is relatively simple.	Location of the metal particles (surface vs. bulk). Clogging or filling the nanosized pores is possible. Electrochemical reduction requires a potentiostat.
Electrodeposition–annealing	Alloy of suitable composition containing the desired metal.	Added metal is part of the nanoporous framework, rather than located on the surface as a single entity. Provides an avenue for synergistic effects since the metals (e.g., Au and Pt) are intimately mixed.	Requires the use of a potentiostat and a furnace. More complex, multistep procedure.
UPD-surface-limited redox replacement	Metal able to undergo UPD. Common examples include Cu and Pb.	Sub-monolayer and monolayer formation. Nanopore framework can be easily maintained. Can be used to convert the NPG scaffold to that of another metal.	Requires the use of a potentiostat. More complex.

**Table 3:** Examples of roughness factors of NPG determined electrochemically.

Substrate	Roughness Factor	Method	References
Gold slide	3–5	Template (-Polystyrene)	[34,59]
Gold slide	20.6	Template (-Silica)	[64]
Steel mold	88.6	Template (-Alumina)	[153]
Gold slide–gold leaf	~12–25	Chemical dealloying	[34,80]
Gold slide–sputtered alloy	2.4–9.3	Chemical dealloying	[154]
Gold–glassy carbon; electrodeposited alloy	4.2–6.5	Electrochemical dealloying	[137]
Gold microwire	27.7	Electrochemically alloying–dealloying	[155]
Gold microwire	86–115	Electrochemically alloying–dealloying	[87]
Gold wire	55–560	Electrochemically alloying–dealloying	[69]
Gold wire	18–213	Electrochemically alloying–dealloying	[70]
Gold rod	280–1020	Anodization, buffer	[98]
Gold CD-R	17.8	Anodization	[93]
Gold disk	43.7	Anodization	[156]
Gold disk	34	Anodization, square-wave pulse, NaOH	[99]

**Table 4:** Examples of non-enzymatic electrochemical sensors based on NPG–metal composites for hydrogen peroxide detection and relevant figures of merit.

Electrode	Linear Range (mM)	LOD (µM)	Sensitivity, μAcm^−2^mM^−1^	Interference Study	Biofouling Test	Ref.
NP-Pt(Au)	0.0709 to 1.25	39.3	148	Yes	Yes	[174]
NPG/PtNPs	0.001 to 0.005	0.0003		Yes	No	[87]
Pt NPs/NPG	10^−4^ to 0.02	0.072		Yes	No	[178]
Au-/nPts	up to ~10	50	264	Yes	No	[153]
NPG/CoO	0.1 to 100	100	62.5	Yes	No	[177]
Co_3_O_4_/(NPG)	0.02 to 19.1	6.4	1338.7	Yes	No	[128]
NPG@Ni foam	0.02 to 9.74	10	2880	Yes	No	[175]

**Table 5:** Examples of non-enzymatic glucose sensors using bimetallic NPG electrodes and relevant figures of merit.

Glucose Sensors	Solution pH	Linear Range, mM	Detection Limit, μM	Sensitivity µAcm^−2^ mM^−1^	Storage Ability	Interference Study	Ref.
NPG-Pt (24%)	Neutral	0.5–10	0.6	145.7	1 month	Yes	[99]
Pd-NPGF	Neutral	1–33	5			yes	[93]
Ni@NPG	Alkaline	1–10^5^ µM		5070.9		No	[140]
Ni(OH)_2_/NPG	Alkaline	0.002–7	0.73	3529	3 weeks	Yes	[126]
CoOx/NPG	Alkaline	0.002–2	0.094	2025	3 weeks	Yes	[127]
NPG/NiCo_2_O_4_	Alkaline	0.01–21	1	0.3871		Yes	[88]
NPG/Co_3_O_4_	Alkaline		0.005	12.5		Yes	[130]
Cu/NPG	Alkaline	0.002–8.11	0.59	3643	>3 weeks	Yes	[123]
NPG/CuO	Alkaline	Up to 12	2.8	374		Yes	[122]
NPG/CoO	Alkaline	Up to 100				Yes	[177]
PtCo/NPG/GP	Alkaline	0.035–30	5	7.84		Yes	[183]
Cu-NPG/SPE	Synthetic saliva, pH 7.5	10^−3^–13	0.13	659.9		Yes	[124]
NiCo-MOF/NPG	Alkaline	0.001–8	0.29	684.4		Yes	[184]
Co_3_O_4_/NPG	Alkaline	0.002–2.1	0.085	4470.4		Yes	[128]

SPE—screen-printed electrode; MOF—metal–organic framework; GP—graphene paper.

## Data Availability

No new data were created or analyzed in this study. Data sharing is not applicable to this article.
